# Photoactivated antifungal polymers prepared by PET-RAFT polymerization

**DOI:** 10.1039/d5sc08534a

**Published:** 2025-12-03

**Authors:** Hatu Gmedhin, Md Aquib, Nathaniel Corrigan, Megan D. Lenardon, Cyrille Boyer

**Affiliations:** a Cluster for Advanced Macromolecular Design (CAMD), Australian Centre for NanoMedicine (ACN), School of Chemical Engineering, UNSW Sydney NSW 2052 Australia cboyer@unsw.edu.au; b School of Biotechnology and Biomolecular Sciences, UNSW Sydney New South Wales 2052 Australia m.lenardon@unsw.edu.au; c Centre for Advanced Manufacturing Technology (CfAMT), School of Engineering, Design and Built Environment, Western Sydney University Sydney NSW 2747 Australia

## Abstract

Rising invasive fungal infections necessitate the rapid development of new, highly efficient antifungal platforms with proven biocompatibility. We report a new class of photoactivated antifungal polymers that incorporate cationic, hydrophobic, and hydrophilic functionalities with a highly efficient photosensitizer. Our approach relies on the use of an acrylate-functionalized zinc(ii)-tetraphenylporphyrin (acryl-ZnTPP) monomer, that performs a dual role: it acts as both the photocatalyst for PET-RAFT polymerization, enabling controlled polymerization, and as an embedded photosensitizer capable of generating reactive oxygen species (ROS) upon light irradiation. Under green or red-light exposure, these polymers show 4–8-fold lower minimum inhibitory concentrations (MICs) and up to 8-fold lower minimum fungicidal concentrations (MFCs) against diverse *Candida* species compared with control polymers (*i.e.*, without acryl-ZnTPP). Hemolysis assays confirm excellent hemocompatibility of these polymers containing ZnTPP as photosensitizer. This approach offers tunable, light-enhanced antifungal activity, providing a promising strategy to combat fungal infections.

## Abstract 2

Rising invasive fungal infections necessitate the rapid development of new, highly efficient antifungal platforms with proven biocompatibility.

## Graphical abstract

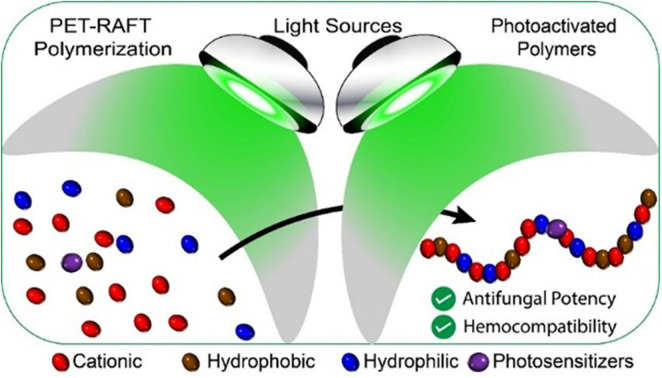

## Introduction

Invasive fungal infections represent a critical and growing global health threat.^1–3^ Each year, more than a billion people are affected, with approximately 6.5 million developing invasive disease that results in over 3.7 million deaths.^4,5^ Among the most concerning pathogens is *Candida*, which causes infections with mortality rates exceeding 35% even with treatment.^6–8^ The growing population of immunocompromised individuals, including patients with HIV/AIDS, cancer, or organ transplants, further amplifies this threat.^9,10^ Despite the growing global burden, therapeutic options for invasive fungal infections remain extremely limited.^11,12^ The few available agents, though clinically valuable, are increasingly compromised by drug resistance, poor bioavailability, and dose-limiting toxicity,^13,14^ which collectively reduce efficacy and contribute to relapse and treatment failure.^15,16^

Host defense peptides (HDPs) have attracted considerable interest as antifungal agents due to their broad-spectrum activity and membrane-disruptive mode of action.^17–19^ However, their clinical translation is hindered by poor pharmacokinetics and limited *in vivo* stability.^20^ To overcome these challenges, synthetic HDP-mimetic antimicrobial polymers have emerged as promising alternatives for combating drug-resistant pathogens.^21–30^ These polymers combine enhanced physicochemical stability, resistance to enzymatic degradation, and high design versatility, enabling precise tuning of charge, hydrophobicity, and functionality.^31–34^ Moreover, their architecture can be engineered to introduce stimuli-responsive or multifunctional behaviors, broadening their therapeutic utility.^35–37^

In parallel, photodynamic therapy (PDT) has emerged as a potent alternative antifungal approach.^38–42^ PDT relies on photosensitizers (PSs) that, upon light activation, generate reactive oxygen species (ROS).^43–45^ ROS oxidatively damage lipids, proteins, and nucleic acids, leading to cell death.^46–48^ Among these PS, water-soluble porphyrins have shown potent antifungal activity, particularly against *Candida* species.^49,50^ However, conventional PDT typically requires high light intensities or elevated PS concentrations to achieve effective ROS generation.^51,52^ Under such conditions, PS, especially planar aromatic porphyrins, tend to aggregate in aqueous environments due to π–π stacking and hydrophobic interactions.^53,54^ This aggregation severely reduces their photodynamic efficacy by quenching excited states, diminishing intersystem crossing efficiency, and limiting singlet oxygen production.^55^ Moreover, aggregated PS often display reduced solubility, altered absorption spectra, and non-uniform cellular uptake, all of which restrict their therapeutic performance and clinical applicability.^56,57^

**Fig. 1:**
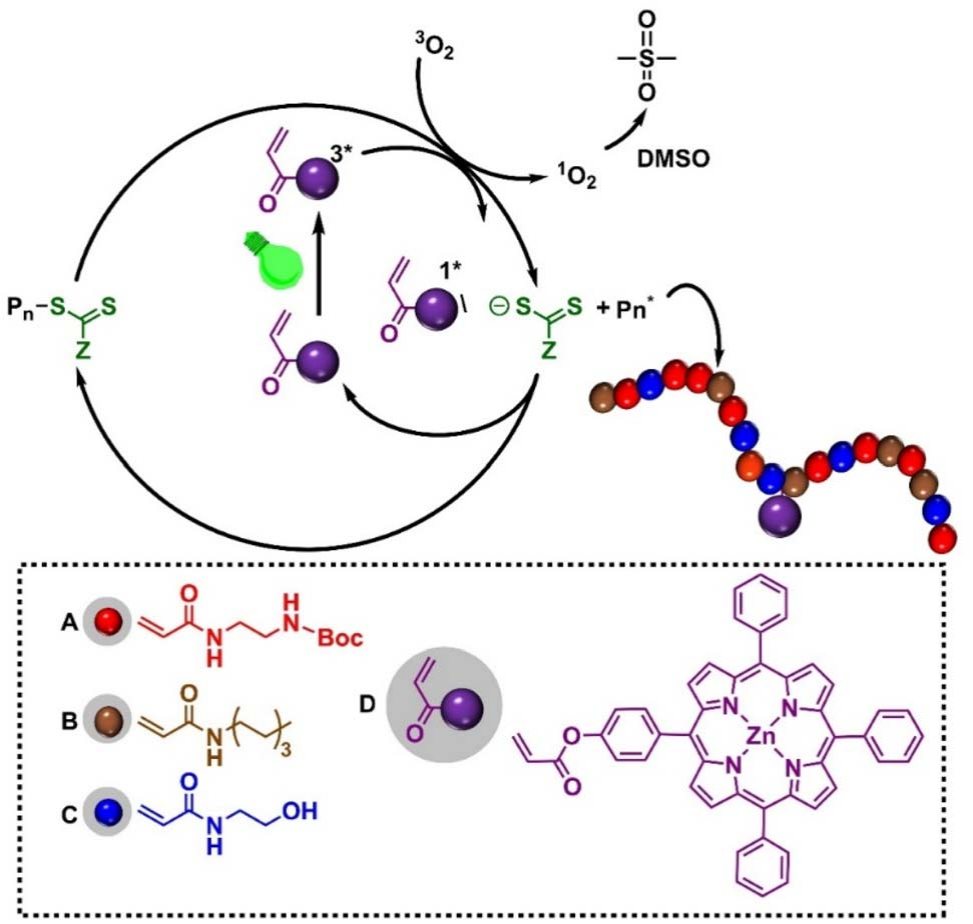
Schematic of proposed mechanism of PET-RAFT polymerization and chemical structures of monomers employed in this study, including protected cationic group Boc-AEm (A, red), hydrophobic monomer HepAm (B, brown), hydrophilic monomer HEAm (C, blue), and photocatalytic monomer acryl-ZnTPP (D, purple).

These limitations underscore the need for an approach that merges HDP-mimetic activity with photodynamic functionality within a single polymeric platform.^58–60^ Recent advances in monomer design featuring wavelength-selective photochemistry and in the incorporation of light-responsive motifs into polymer backbones have now made this possible.^61–64^ Here, we introduce a new class of photoactive antifungal polymers that integrate HDP-like properties with light-triggered ROS generation. The polymers were synthesized *via* photoinduced electron/energy transfer–reversible addition–fragmentation chain transfer (PET-RAFT) polymerization, incorporating a zinc(ii)–tetraphenylporphyrin acrylate (acryl-ZnTPP) monomer that serves both as the photocatalyst for PET-RAFT and as an embedded photosensitizer.^65–67^ The resulting copolymer, P(ABCD), comprises four functional components: a cationic group (A), a hydrophobic group (B), a neutral hydrophilic group (C), and an acryl-ZnTPP moiety (D) capable of generating ROS upon visible-light irradiation (Fig. 1). This design preserves the intrinsic antifungal efficacy of HDP-mimetic polymers while enabling on-demand, localized photodynamic activation, thereby enhancing therapeutic selectivity and minimizing off-target effects.

## Results and discussion

### Photoactive polymer synthesis

The design of our photoactive polymer was based on prior work with a polyacrylamide terpolymer with potent antifungal activity and biocompatibility.^22,32,68^ Four types of monomers were employed: (A) a cationic Boc-protected *tert*-butyl (2-acrylamidoethyl) carbamate (Boc-AEm), deprotected post polymerization to yield a primary ammonium group for electrostatic interactions with fungal membranes,^21^ (B) hydrophobic *N*-heptyl acrylamide (HepAm) to promote membrane interaction,^22^ (C) hydrophilic *N*-hydroxyethyl acrylamide (HEAm) to enhance solubility and biocompatibility,^69^ and (D) an acrylate functionalized zinc tetraphenyl porphyrin (acryl-ZnTPP) to enable light-activated ROS generation (see SI, Fig. S1–S4). Both Boc-AEm and HepAm were synthesized using carbodiimide-mediated amine coupling (SI, Scheme S1),^32^ whereas HEAm was used as received. The photoactive acryl-ZnTPP monomer was prepared by acrylate functionalized zinc tetraphenyl porphyrin, followed by zinc metalation (SI, Scheme S2).^70^

Polymers were synthesized *via* one-pot PET-RAFT polymerization (SI, Scheme S3). P(ABCD) was achieved by incorporating the vinyl-functionalized acryl-ZnTPP monomer into the polymer backbone in a 1 : 1 ratio with the chain transfer agent 2-(butylthiocarbonothioylthio)propanoic acid (BTPA; SI, Fig. S5). This molar ratio was selected as an optimized compromise to achieve two critical objectives: (1) to provide sufficient photocatalytic activity for effective PET-RAFT polymerization, and (2) to preserve key polymer properties, particularly water solubility. Because the ZnTPP moiety is hydrophobic and can markedly reduce polymer solubility in aqueous media, its incorporation was intentionally kept minimal.

**Fig. 2:**
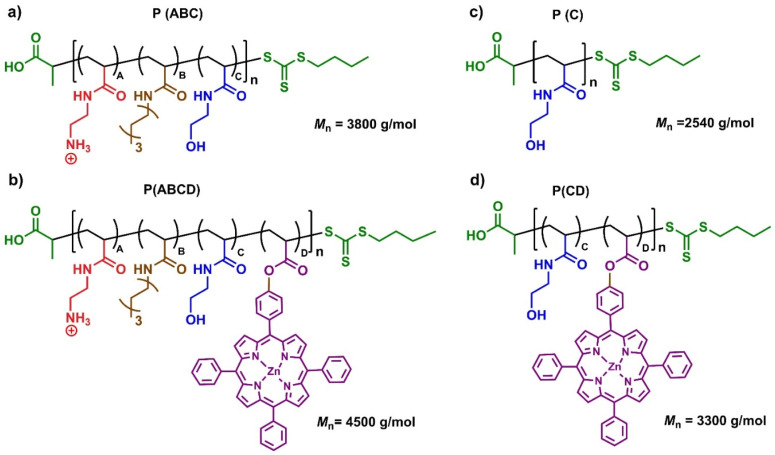
Chemical structures of polymers: (a) P(ABC), an antifungal terpolymer containing AEm (A, red), HepAm (B, brown), and HEAm (C, blue); (b) P(ABCD), a photoactive analogue of P(ABC) containing acryl-ZnTPP (D, purple); (c) P(C), HEAm homopolymer used as a control; and (d) P(CD), a statistical copolymer of HEAm (C) and acryl-ZnTPP (D) for assessing light responsiveness.

To validate the specific photoactivity of the P(ABCD), two control systems were synthesized under identical conditions (Fig. 1). The first, referred to as the P(ABC) terpolymer, was synthesized using Zinc(ii)–tetraphenylporphyrin (ZnTPP) as an external photocatalyst, followed by repeated precipitation (three times in a ratio of *n*-hexane : diethyl ether, 4 : 1) to remove ZnTPP, ensuring no residual PS remained in the polymer.^70^ The second control group comprised two non-antifungal polymers, a homopolymer of HEAm and a HEAm-*stat*-acryl-ZnTPP copolymer, containing similar amount of acryl-ZnTPP in P(ABCD). These controls were designed to assess the photodynamic activity of P(ABCD) (Fig. 2).

### Polymer characterization

A variety of analytical techniques, including spectroscopy and chromatography, were used to confirm the precise chemical structure, composition, and successful incorporation of acryl-ZnTPP PS into the polymer. Firstly, ^1^H NMR spectroscopy revealed monomer conversions exceeding 99%, as evidenced by the disappearance of the vinyl proton signals, which range from *δ* = 5.5 to 6.3 ppm (SI, Fig. S6). The purified copolymer backbone displayed distinct signals corresponding to the incorporated functional monomers, for example, hydroxyl group of HEAm (*δ* = 4.5 ppm), aliphatic protons of HepAm (*δ* = 0.8–1.5 ppm), Boc-AEm amide protons range from *δ* = 6.5–7.5 ppm, and aromatic signals of acryl-ZnTPP (*δ* = 7.9–8.2 ppm) (SI, Fig. S7 and S8). The close agreement between the feed and experimental ratios confirmed the successful incorporation of functional monomeric units (Table 1 and SI, Fig. S8). Further, evaluation of polymerization kinetics and copolymer composition at different time points demonstrated the statistical incorporation of these monomers (SI, Fig. S11). Diffusion-order NMR spectroscopy (DOSY) provided further confirmation of the successful incorporation of acryl-ZnTPP (monomer D) into P(ABCD) polymer and the complete absence of residual unreacted monomer. This was evidenced by the identical diffusion coefficient (*D* ∼ 10^−7^ cm^2^ s^−1^) measured for the porphyrin signals and the polymer backbone protons, corresponding to a single, high-molecular-weight species (SI, Fig. S12).

**Table 1:** Characterization of the polymers synthesized in this study

Polymers	*M*_*n*_ a (kg mol^−1^)	NMR			SEC		DLS^f^	
		*X*_*n*_ NMR^b^	*M*_*n*_ c (kg mol^−1^)	Composition: A : B : C : D^d^	*M*_*n*_ e (kg mol^−1^)	*Đ* f	*D*_h_ (nm)	*ζ* (mV)
P(ABC)	3.8	20	3.8	50 : 26 : 24 : 0	6.2	1.10	1.5	55.3
P(ABCD)	4.5	21	4.5	49 : 26 : 25 : 1	6.8	1.12	5.9	36.4
P(C)	2.5	20	2.5	0 : 0 : 100 : 0	6.5	1.09	3.2	−20.3
P(CD)	3.3	21	3.4	0 : 0 : 100 : 1.2	7.5	1.14	2.5	−27.8

a The target molecular weights were calculated based on the feed ratios of the reactants.

b The degree of polymerization (*X*_*n*_) was calculated using ^1^H NMR (SI, Fig. S6).

c The *M*_*n*_ was calculated using ^1^H NMR (SI, Fig. S7).

d The composition ratio was calculated by ^1^H NMR (SI, Fig. S8).

e *M*_*n*_ of the Boc-protected terpolymer was determined by SEC, using *N*,*N*′-dimethylacetamide as eluent and PMMA standards (200–10^6^ g mol^−1^).

f Dispersity (*Đ*) was determined using size-exclusion chromatography (SEC) analysis. The *D*_h_ (hydrodynamic diameter in nm) and *ζ* (zeta potential in mV) were determined using dynamic light scattering (DLS).

**Fig. 3:**
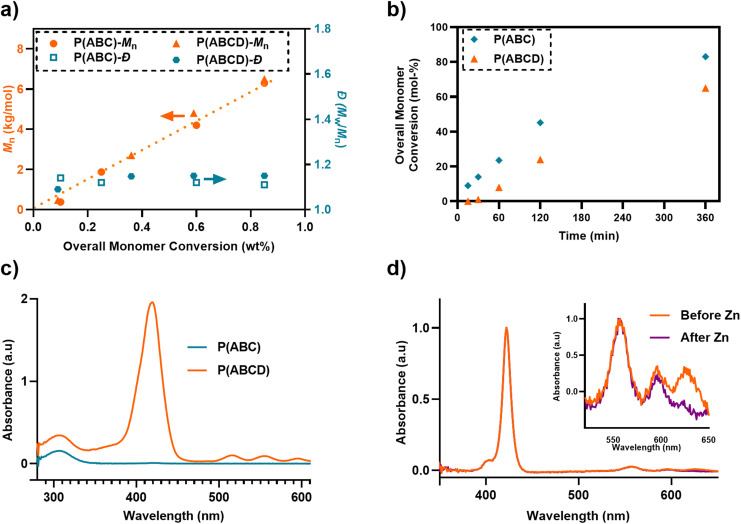
Characterizations of P(ABC) and photoactive P(ABCD) polymers: (a) MWD and dispersity (*Đ*) *versus* function of mass-based overall monomer conversion; (b) overall monomer conversion *versus* time; (c) UV-vis spectra of P(ABC) and P(ABCD) in RPMI-1640 media; and (d) absorbance spectra of polymer P(ABCD) after TFA treatment, but before (orange) and after (aqua) zinc metalation.

Size-exclusion chromatography (SEC) confirmed successfully controlled polymerization, with number-average molecular weights (*M*_*n*_) ranging from 6.2–7.5 kg mol^−1^ and low dispersity (*Đ*) values between 1.08–1.16 (Fig. 3a and Table 1). All polymers exhibited a narrow molecular weight distribution (MWD), however, a slight increase in *Đ* was observed in the copolymer incorporating acryl-ZnTPP monomer (Table 1, P(C), *Đ* = 1.14), consistent with previous reports.^61^ However, a divergence between theoretical *M*_*n*_ (P(ABC) ∼ 3.8 kg mol^−1^ and P(ABCD) ∼ 4.5 kg mol^−1^) and SEC-derived experimental *M*_*n*_ (P(ABC) ∼ 6.2 kg mol^−1^ and P(ABCD) ∼ 6.8 kg mol^−1^) were observed, which was attributed to the difference in hydrodynamic volume between amphiphilic terpolymers and polymethyl methacrylate (PMMA) standard in *N*,*N*′-dimethylacetamide eluent.^32,71^

### Polymer deprotection and metallization

Deprotection of Boc-groups in both P(ABC) and P(ABCD) was achieved *via* trifluoroacetic acid (TFA) and confirmed by the complete disappearance of the *tert*-butyl proton signal at *δ* = 1.4 ppm in the ^1^H NMR spectra (SI, Fig. S9). However, after this treatment, we observed that zinc was removed (Fig. 3d and SI, Fig. S10a). Subsequently, polymers bearing acryl-TPP moieties were treated with zinc acetate to reintroduce zinc in the porphyrin. Successful incorporation of zinc was confirmed by the emergence of ZnTPP-specific absorption bands and the corresponding disappearance of free-base porphyrin peaks in the UV-vis spectrum (Fig. 3d and SI, Fig. S10b). In addition, UV-SEC analysis (Size-Exclusion Chromatography with a UV detector at 420 nm, corresponding to the Soret absorption band of the ZnTPP unit) was performed on P(ABCD), acryl-ZnTPP (monomer D), and P(ABC). P(ABCD) exhibits a clear UV absorption signal at 420 nm at a short elution time, unambiguously confirming the incorporation of monomer D into the polymer chain. In contrast, P(ABC) shows no detectable signal at 420 nm, consistent with the absence of the porphyrin unit. The free monomer D displays a distinct UV-SEC trace at a substantially longer elution time, reflecting its much lower molecular weight (SI, Fig. S13).

### Aqueous media characterization

UV-Vis absorbance spectra of P(ABCD) in aqueous RPMI-1640 medium exhibited characteristic porphyrin absorbance bands, including the Soret band at ∼425 nm and Q-bands at ∼550 and ∼600 nm, consistent with the spectral fingerprint of acryl-ZnTPP (Fig. 3e). These signals were absent from the P(ABC), confirming the successful incorporation of the photosensitizer into P(ABCD). Dynamic light scattering (DLS) analysis revealed that the polymer synthesized in this study has a hydrodynamic diameter of less than 10 nm (Table 1). Additionally, zeta potential (*ζ*) measurements for P(C) and P(CD) polymers showed a negative net charge in the Milli-Q water (P(C) = −20.3 and P(CD) = −27.8 mV; Table 1). However, net positive surface charge was observed following Boc-group removal for P(ABC) = +55 and P(ABCD) = +36 (Table 1), confirming successful deprotection and the generation of primary ammonium functionalities.^22^

### Antifungal activity and light-responsive enhancement

The antifungal activity of the synthesized polymers was evaluated by determining the minimum inhibitory concentrations (MICs) according to the Clinical and Laboratory Standards Institute (CLSI) guidelines, as described previously.^22,31,72^ The antifungal susceptibility test was performed against three *Candida* species: the reference strain *C. albicans* (SC5314),^73^ a clinical isolate of *C. albicans* (b30708/5)^74^ with reduced susceptibility to pristine polymer P(ABC),^21,32^ and a type strain of *C. parapsilosis* (ATCC22019).^75^ Conventional antifungal agents, fluconazole (Flu) and amphotericin B (Amp-B), were included as positive controls; both agents demonstrated consistent susceptibility profiles under standard conditions (Table 2).^22,31,32^ Green light (*λ*_max_ ≈ 530 nm) or red light (*λ*_max_ ≈ 630 nm) in 96-well plate LED setups was used to activate acryl-ZnTPP, incorporating polymers, as these wavelengths correspond to the Q-bands of acryl-ZnTPP (SI, Fig. S14 and S15).

**Table 2:** MIC_90_ of synthesized polymers without irradiation and upon green or red-light irradiation at 3.45 mW cm^−2^ for a total exposure time of 60 min^a^

Fungal species	Irradiation													
	P(ABC)			P(ABCD)			P(C)			P(CD)			Flu	AmpB
	None	Green	Red	None	Green	Red	None	Green	Red	None	Green	Red	None	None
*C. albicans* (SC5314)	32	32	32	32–64	4–8	16	>512	>512	>512	>512	256	512	0.25	1
*C. albicans* (b30708/5)	256	256	256	256–512	32	32–64	>512	>512	>512	>512	256	512	0.25	0.5–1
*C. parapsilosis* (ATCC 22019)	128	128	128	128	16	32	>512	>512	>512	>512	256	512	2–4	1

a The MIC_90_ (µg mL^−1^) values represent the minimum inhibitory concentration required to inhibit at least 90% of *Candida* species growth. Control assays were performed under identical conditions in flat-bottom 96-well, non-pyrogenic, polyester microplates and illuminated with a custom-designed red and green LED panel (at a consistent 13 V to the red and green source and measured a green-light intensity of 3.45 mW cm^−2^ at the surface of the assay) integrated into a 96-well plate illumination system, with a maximum exposure time of 60 min. Antifungal drugs fluconazole (Flu) and amphotericin B (AmpB) were included as reference controls.

Initially, control assays conducted without irradiation confirmed that both P(ABC) and P(ABCD) exhibited potent antifungal activity with no noticeable difference in MIC values (Table 2). This indicates that incorporating acryl-ZnTPP as a functional monomer at a 1 : 1 ratio to BTPA RAFT agents (P(ABCD)) does not compromise the inherent antifungal properties of P(ABC). Upon irradiation (3.45 mW cm^−2^, 60 min) with green (*λ*_max_ ≈ 530 nm) or red light (*λ*_max_ ≈ 630 nm), P(ABCD) displayed an enhanced antifungal activity (MIC = 4–8 µg mL^−1^), whereas P(ABC) showed no change (MIC = 32 µg mL). As expected, P(C) and P(CD) were inactive without irradiation (MIC > 512 µg mL^−1^); following irradiation, P(CD) exhibited weak antifungal activity (MIC = 256 to 512 µg mL^−1^), whereas P(C) remained inactive (MIC >512 µg mL^−1^). These results clearly demonstrate that the incorporation of acryl-ZnTPP into the polymer reduces MICs by 4–8-fold, conferring light-responsive antifungal activity. Both green and red light successfully enhanced the antifungal performance of P(ABCD), however, green light provided the lowest MIC values. Therefore, in the next part of this study, we selected green light in subsequent experiments because acryl-ZnTPP absorbs more strongly in the green than in the red at matched power (SI, Fig. S15), which maximizes photosensitizer excitation and ROS generation, proving a more sensitive test of photo-triggered antifungal activity.

**Fig. 4:**
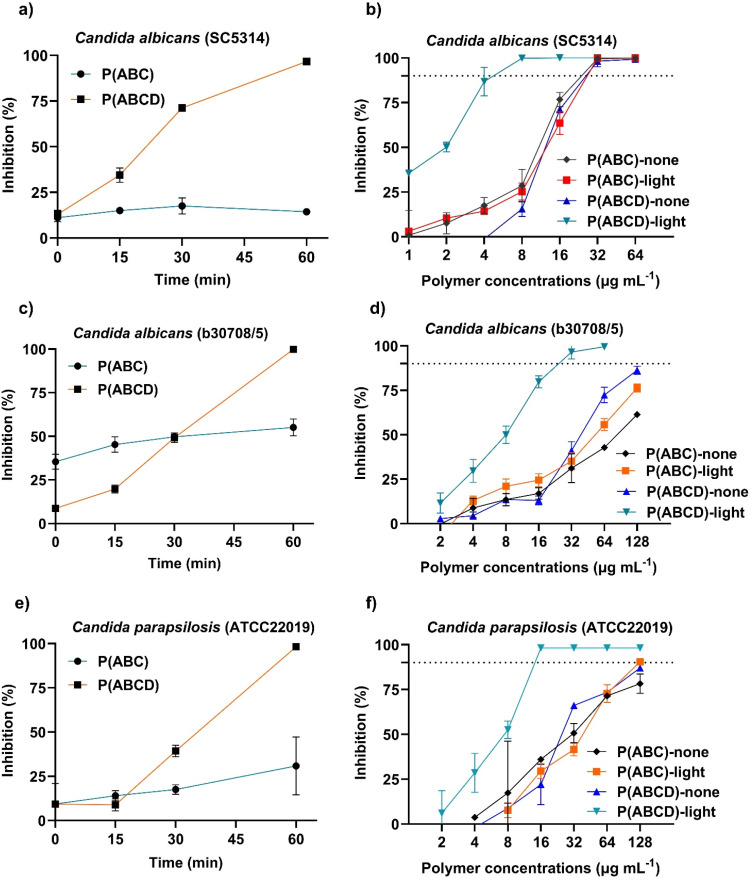
Kinetic growth inhibition of *Candida* species treated with P(ABC) and P(ABCD). Time-dependent (a), (c) and (e) and polymer concentration-dependent (b), (d) and (f) growth inhibition (%) for (a) and (b) the laboratory strain *C. albicans* (SC5314), for (c) and (d) the clinical isolate: *Candida albicans* (b30708/5), and for (e) and (f) *C. parapsilosis* (ATCC22019). In panels a, c, and e, polymers were added at a fixed concentration of 16 µg mL^−1^, and plates were irradiated with green light (*λ*_max_ ≈ 530 nm, at 3.45 mW cm^−2^ at the assay surface) for 15, 30, and 60 min. In panels b, d, and f, polymers were added at the concentrations indicated and plates were irradiated with green light (*λ*_max_ ≈ 530 nm, at 3.45 mW cm^−2^ at the assay surface) for 60 min. Non-irradiated plates served as controls. The horizontal dashed line indicates the minimum inhibitory concentration that achieves 90% inhibition (MIC_90_). In all panels, growth inhibition (%) was calculated relative to negative control wells. All tests were performed in triplicate, and the mean values are presented with error bars representing the standard deviation.

To exclude the possibility that light alone contributed to antifungal effects, untreated fungal cultures (no polymers) were exposed to green light (3.45 mW cm^−2^) under identical conditions for 60 min. Optical density measurement at 600 nm (OD_600_) after 24 h of incubation showed no significant differences between irradiated and non-irradiated controls (SI, Fig. S16), confirming that the applied light did not induce detectable photodamage. Building on this, the kinetics of light-responsive antifungal activity were assessed using time-dependent growth inhibition assays under green light.^49^ Fungal cultures were treated with a fixed concentration (16 µg mL^−1^) of either P(ABC) or P(ABCD) and exposed to green light (*λ*_max_ ≈ 530 nm, 3.45 mW cm^−2^) for 0, 15, 30, and 60 min. P(ABCD)-treated groups exhibited a pronounced, time-dependent antifungal response, with significant growth inhibition at 30 min and complete inhibition (MIC >90%) at 60 min (Fig. 4). When combined with the time-dependent results shown in Fig. 4a, c and e, this finding suggests that sufficient photoinduced reactive oxygen species are generated during 1 h of irradiation at 16 µg mL^−1^ of the polymer to kill fungal cells. In contrast, P(ABC) showed only moderate growth inhibition, with no MIC achieved even after 60 min of irradiation. These data indicate that growth inhibition increases as the time of exposure of P(ABCD) to green light increases.

In addition to growth inhibition studies, the fungicidal activity of the polymers was assessed by determining their minimum fungicidal concentration (MFC).^76^ Without irradiation, MFC values were typically 4–8 times higher than the corresponding MICs, indicating that higher concentrations were required to achieve complete fungal eradication, as previously reported (Table 3).^31,32^ Upon light irradiation, however, MFC values decreased to roughly twice the corresponding MICs (Table 3), demonstrating that photoactivation amplifies the fungicidal activity of P(ABCD). This enhancement likely arises from light-triggered ROS generation, complementing the antifungal activity of the polymer, resulting in more effective fungicidal properties. The observed light-enhanced fungicidal responses could be valuable for managing invasive fungal infections, where complete eradication is crucial preventing recurrence and the development of resistance.^51,77^

**Table 3:** Fungicidal and hemolytic activity of polymers synthesized in this study and antifungal drugs^a^

Polymers and antifungal drugs	MFC						HC_50_
	*C. albicans* (SC5314)		*C. albicans* (b30708/5)		*C. parapsilosis* (ATCC 22019)		
	Irradiation		Irradiation		Irradiation		
	−	+	−	+	−	+	
P(ABC)	256	256	>512	>512	>512	>512	>2000
P(ABCD)	256	16–32	>512	32–64	>512	32	>2000
P(C)	>512	>512	>512	>512	>512	>512	>2000
P(CD)	>512	512	>512	512	>512	256–512	>2000
Amp-B	>2	>4	2	>4	2	>4	18
Flu	>4	>4	>4	>4	>4	>4	>125

a Minimum fungicidal concentration (MFC, 48 h, µg mL^−1^) determined as the lowest concentration at which no visible fungal growth was observed after 48 h of incubation at 30 °C, by subculturing samples from MIC assay wells. The assays, marked as (+), were performed under green light (3.45 mW cm^−2^) for 60 min. HC_50_ corresponds to the polymer concentration that induces 50% hemolysis of sheep red blood cells after 2 h of incubation. Hemolysis assay conducted under standard conditions (no irradiation) up to a maximum concentration of 2000 µg mL^−1^.

Building on the demonstrated biocompatibility of the antifungal polymer, hemocompatibility was examined to evaluate the potential impact of PS incorporation.^21,22,31,32^ Hemolytic activity was measured against defibrinated sheep red blood cells (RBCs) under standard conditions (no irradiation; see SI).^35^ All tested polymers, including P(ABC) and P(ABCD), exhibited minimal hemolytic activity, with HC_50_ values exceeding the highest concentration tested (>2000 µg mL^−1^; Table 3). These results indicate that incorporation of PS does not compromise the hemocompatibility of the polymers.

**Fig. 5:**
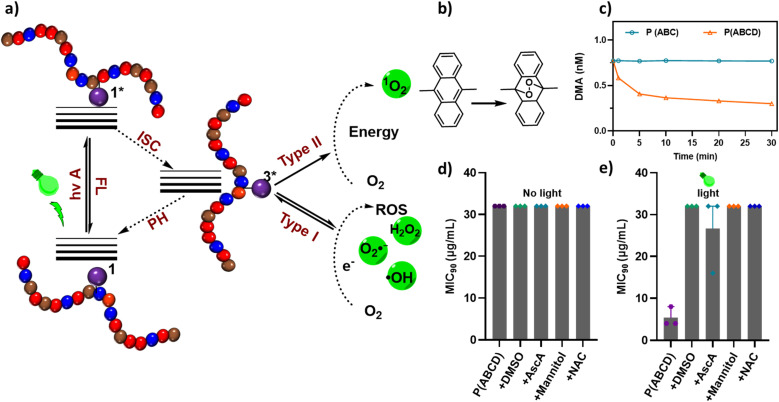
Dual-action antifungal mechanisms. (a) Schematic illustration of reactive oxygen species (ROS) generation pathways. (b) Reaction of 9,10-dimethylanthracene (DMA) as a singlet oxygen (^1^O_2_) trap, producing a stable endoperoxide. (c) Time-dependent quenching of DMA in the presence of polymers P(ABC) and P(ABCD) under light irradiation. (d) MIC values of P(ABCD) in the presence of ROS scavengers under dark conditions, and (e) MIC values of P(ABCD) with the ROS scavengers under green light irradiation (*λ*_max_ ≈ 530 nm, 3.45 mW cm^−2^) for 60 min. MIC_90_ values in panels (c)–(e) are the average of three replicates, with error bars indicating the minimum-to-maximum range.

To elucidate the mechanism underlying the observed light-enhanced fungicidal effects, we investigated the type of generated ROS by the acryl-ZnTPP-functionalized copolymer. Metalated porphyrins are well-known for their ability to efficiently produce singlet oxygen (^1^O_2_) and other ROS upon photoactivation.^78–80^ This process involves direct energy transfer from the excited PS to molecular oxygen, converting it from the ground triple state (^3^Σg^−^) to an excited single state (^1^Δg or ^1^Σg^+^).^81^ Singlet oxygen generation was first examined using 9,10-dimethylanthracene (DMA) as a chemical trap in dimethylformamide. A light-dependent decrease in DMA absorbance confirmed that P(ABCD) effectively produced ^1^O_2_ (Fig. 5b).^61^ In addition to ^1^O_2_, other ROS can be produced by PS, contributing to antifungal activity.^82^ To probe the involvement of other specific ROS species, we conducted scavenger assays employing ascorbic acid (AscA), dimethyl sulfoxide (DMSO), mannitol, and *N*-acetylcysteine (NAC), each known to quench distinct ROS types (SI, Table S1).^49^

In the presence of DMSO, mannitol, or NAC, the MIC increased approximately two-fold, indicating the participation of singlet oxygen (^1^O_2_), hydroxyl radical (OH˙), and superoxide anions (O_2_˙^−^) in fungal killing (Fig. 5b–f). Furthermore, the addition of AscA, a well-established singlet oxygen quencher that can yield H_2_O_2_ and OH˙ upon oxidation, further elevated the MIC, underscoring the critical contribution of ^1^O_2_ to the photodynamic mechanism, although the effect did not fully reach the level observed under dark conditions (Fig. 5c and SI Table S1).^83^ These results confirm that singlet oxygen plays a predominant role in the photo-activated antifungal activity of P(ABCD). Overall, these results demonstrate that the acryl-ZnTPP units embedded within the polymer backbone act as efficient PS, primarily operating *via* a type I pathway to generate ^1^O_2_ through a triplet–triplet annihilation (TTA) mechanism.^84^

Evidence of light-activated ROS generation confirms an additional mechanism of action beyond that previously described for the antifungal polymers P(ABC), thereby enhancing their fungicidal efficacy. This strategy eradicated planktonic fungal cells, including a *C. albicans* clinical isolate exhibiting reduced susceptibility to P(ABC) (Tables 2 and 3). Mechanistically, previous studies have shown that ROS-induced oxidative stress disrupts fungal membrane integrity, thereby facilitating deeper ROS penetration and amplifying the fungicidal response.^85^

## Conclusions

In this study, we developed a new class of photoactivated antifungal polymers that integrate peptide-mimetic terpolymer segments with a vinyl-functional ZnTPP-based photosensitizer (PS), incorporated directly during polymerization. This one-step approach enables the synthesis of intrinsically photoactive polymers with a statistical distribution of PS units along the backbone, eliminating the need for post-synthetic modification. The resulting polymers exhibited strong antifungal activity against *C. albicans* and *C. parapsilosis*, with markedly enhanced potency under light activation compared to the control polymer P(ABC). Embedding the PS within the polymer chain provides both spatial and temporal control over activity, offering a versatile strategy for treating drug-resistant fungal infections.

This work provides initial *in vitro* evidence that incorporating PS into antimicrobial polymers can substantially enhance antifungal performance, suggesting potential benefits in applications where external light delivery is feasible. However, further studies are required to assess their activity and safety in more complex biological environments. Overall, these polymers warrant deeper investigation against resistant clinical isolates, within biofilm models, and under physiologically relevant conditions. Moreover, the acryl-ZnTPP polymers present a tunable platform in which PS content can be systematically optimized, offering significant potential for advanced antifungal therapies, particularly when paired with emerging light-delivery technologies.

## Author contributions

Hatu Gmedhin: conceptualization, methodology, investigation, formal analysis, writing – original draft. Md Aquib: writing – review & editing. Nathaniel Corrigan: validation, writing – review & editing. Megan D. Lenardon: validating, resources, writing – review & editing. Cyrille Boyer: conceptualization, resources, writing and editing – review & editing, supervision.

## Conflicts of interest

The authors declare no competing financial interest.

## Supplementary Material

SC-017-D5SC08534A-s001

## Data Availability

The data supporting this article have been included as part of the supplementary information (SI). Supplementary information: Schemes S1–S4, Fig. S1–S16, Table S1, and the Experimental methods and Materials sections. See DOI: https://doi.org/10.1039/d5sc08534a.
