# Supplementary material for: Photoactivated antifungal polymers prepared by PET-RAFT polymerization
Source: Chem Sci. 2025 Dec 3;17(6):3178–88. doi: 10.1039/d5sc08534a (PMC12706916; doi:10.1039/d5sc08534a)
Supplement: SC-017-D5SC08534A-s001 [file SC-017-D5SC08534A-s001.pdf]

Supplementary Information (SI) for Chemical Science.
This journal is © The Royal Society of Chemistry 2025

Supporting Information

Photoactivated Antifungal Polymers Prepared by PET-RAFT Polymerization

Hatu Gmedhin[1,2], Md Aquib[1], Nathaniel Corrigan[3], Megan D. Lenardon[2*], Cyrille Boyer[1*]

[1]Cluster for Advanced Macromolecular Design (CAMD) and Australian Centre for NanoMedicine (ACN), School of Chemical Engineering, UNSW, Sydney, NSW, 2052, Australia

[2]School of Biotechnology and Biomolecular Sciences, UNSW, Sydney, New South Wales, 2052, Australia

[3]Centre for Advanced Manufacturing Technology (CfAMT), School of Engineering, Design and Built Environment, Western Sydney University, Sydney, NSW 2747

E-Mails: cboyer@unsw.edu.au; m.lenardon@unsw.edu.au

Contents

Materials and Methods 1
Materials 1
Monomer synthesis 2
Hydrophobic and cationic monomers: 2
Acryl-Zinc(II)-teteraphenylporphyrin (acryl-ZnTPP) *monomer:* 3
Polymer synthesis 5
ZnTPP-activated PET-RAFT polymerization 5
Photoactivated polymers prepared by PET-RAFT polymerization 8
Post-polymerization: 8
Boc-deprotection using trifluoroacetic acid (TFA), 8
Photoactive polymer Re-metallization. 8
Characterizations 9
Biological experiment: 10
Media Preparation: 10
Fungal Strain and Culture Condition: 10
Minimum Inhibitory Concentration (MIC) Assay: 10
Minimum fungicidal concentration (MFC): 11
Hemolytic Assay: 11
Optical setup and photochemical Assay 12
Light source photoreactor setup: 12
Singlet Oxygen Quantification Using DMA Quenching 12
Supporting information - Figures and Tables 15
References 30

Materials and Methods

Materials

1-(3-dimethylaminopropyl)-3-ethyl carbodiimide hydrochloride (EDC, 99%) and tert-butyl (2-aminoethyl) carbamate (97%) were obtained from AmBeed. 4-Hydroxybenzaldehyde (95%), benzaldehyde (99%), Acrylic

acid (AA, 99%) and pyrrole (98%), heptylamine (99%), *N*-hydroxyethyl acrylamide (HEAm, contains 1,000 ppm monomethyl ether hydroquinone as stabilizer, 97%), 5,10,15,20-tetraphenyl-21*H*,23*H*-porphine zinc (ZnTPP), Zinc acetate dihydrate (>98%), dimethylacetamide (DMAc, HPLC grade), 2-(*n*-butylthiocarbonothioylthio) propanoic acid (BTPA, 95%) were purchased from Sigma Aldrich. Trifluoroacetic acid (TFA, 99%), diethyl ether (95%), hexane (95%), ethyl acetate, sodium bicarbonate ($NaHCO_3$), sodium chloride (NaCl), and magnesium sulphate ($MgSO_4$), were supplied by Chem Supply. Deuterated dimethyl sulfoxide (DMSO) was purchased from Cambridge Isotope Laboratories; dichloromethane and acryloyl chloride (>96%) were sourced from Merck. Hydrochloric acid 32% was purchased from RCI Labscan. Biological reagents, including Oxoid™ phosphate-buffered saline tablet (PBS), 3-morpholinopropane-1-sulfonic acid (MOPS), Roswell Park Memorial Institute medium (RPMI-1640 powder), Oxoid Peptone Mycological, Oxoid Yeast Extract Powder, and Agar Technical were all purchased from Thermo Scientific™. Antifungal agents fluconazole (≥98%) and amphotericin B (approx. 80% from *Streptomyces* sp.) were obtained from Sigma Aldrich. D-glucose and Triton™ X-100 were also sourced from Chem Supply. Defibrinated sheep red blood cells (RBCs) were obtained from Serum Australis (Australia). Milli-Q water was acquired obtained from the Sartorius Arium® purification system with a resistivity of 18.2 $m\Omega\ cm^{-1}$.

Monomer synthesis

<u>*Hydrophobic and cationic monomers:*</u>

Hydrophobic monomer *N*-heptyl acrylamide (HepAm) and the cationic monomer *tert*-butyl (2-acrylamidoethyl) carbamate (Boc-AEAm) were synthesized using a modified EDC-mediated coupling protocol **(Scheme S1)**.[1] Briefly, 1.2 equivalents of EDC were dissolved in DCM (1 mmol 2 mL^{-1}) in a 100 mL round-bottom flask under nitrogen gas. The mixture was stirred in an ice bath for 10 min, assigned as step one reaction (rxn-1). Then, in this reaction, acrylic acid (1.2 eq.) was added to activate the carboxylic acid group. This reaction (rxn-1) is common to both products (a → HepAm) and (b → Boc-AEAm).

As shown in **Scheme S1,** HepAm (35.47 mmol of heptylamine, a^1) was dissolved in 35 mL of DCM and added dropwise to reaction (rxn-1), the activated solution containing 1.2 equivalents of acrylic acid, and stirred

overnight at room temperature. The resulting crude mixture was sequentially washed with 1 × 0.1 M HCl, a saturated $NaHCO_3$ solution, brine, and water. The organic phase was dried over anhydrous $MgSO_4$, filtered, and the solvent was removed under reduced pressure. The crude product (a) was purified by flash column chromatography in a 7:3 (v/v) hexane/ethyl acetate mixture to yield the final compound HepAm, as a white, greasy solid ((45%, >98% purity). The purified product was dried under vacuum and characterized by proton nuclear magnetic resonance spectroscopy (^{1}H NMR) (supporting information (**SI, Figure S1**).

Similarly, as shown in Scheme S1, Boc-AEAm was synthesized by dissolving *tert-butyl* (2-aminoethyl) carbamate (46.67 mmol, b^1) in 47 mL DCM and adding dropwise to reaction (rxn-1), which contained the activated solution and 1.2 eq of acrylic acid. The mixture was stirred overnight at room temperature. The resulting crude mixture was sequentially washed three times each with 0.1 M HCl, a saturated $NaHCO_3$ solution, brine, and water. The organic phase was dried over $MgSO_4$, filtered, and concentrated by evaporating DCM. The product (b) was purified by repeated precipitation (4×) in cold diethyl ether and centrifuged at 9000 rpm for 5 min. The purified product, a fine white powder with a 65% yield, was dried in vacuo and characterized by ^{1}H NMR (**SI, Figure S2**).

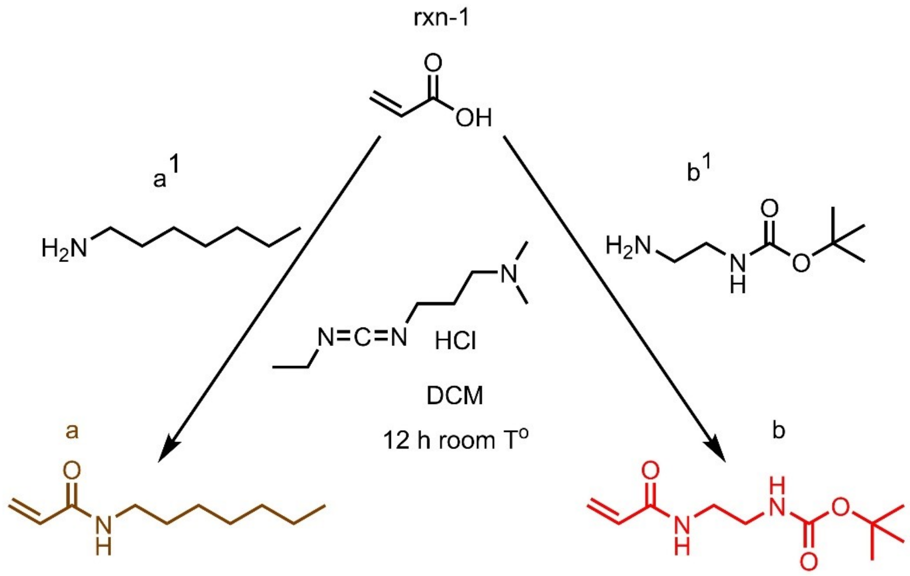

Scheme S1 Synthesis routes of (a) HepAm and (b) Boc-AEAm.

Acryl-Zinc(II)-teteraphenylporphyrin (acryl-ZnTPP) *monomer:*

Acryl-Zinc(II)-teteraphenylporphyrin (acryl-ZnTPP) was synthesized following a previously reported protocol **(Scheme S2).**[2] A solution of 4-hydroxybenzaldehyde (**a,** 36 mmol) and benzaldehyde (**b**, 102 mmol) in propionic acid (500 mL) was heated to near reflux. Pyrrole (**c,** 144 mmol) was added dropwise under stirring. The mixture was refluxed for 1 h, then cooled, and the solvent was removed by ~50% under reduced pressure. Methanol (400 mL) was added, and the solution was stored overnight at 4 °C and then filtered. The precipitate was collected, washed with cold methanol, and dried under vacuum. The crude product was purified by flash column chromatography on silica gel (DCM, isocratic): the third band was collected and concentrated to afford a violet solid, 5-(4-hydroxyphenyl)-10,15,20-triphenylporphyrin (**d**, TPP-OH) (5% yield). For acylation, TPP-OH (1.58 mmol) and TEA (2.6 mmol) were dissolved in DCM. Acryloyl chloride (2.4 mmol) was added dropwise to the solution at 0 °C under nitrogen gas bubbling, and the mixture was stirred for 2 h. The solvent evaporated, and the residue was redissolved in 150 mL (4:1 v/v/; DC/methanol (e). Finally, Zinc acetate dihydrate (18.86 mmol) was added, and the mixture was stirred overnight at room temperature. The solvent was removed under reduced pressure, and the residue was purified by flash column chromatography on silica gel (DCM, isocratic) to give acryl-ZnTPP **(f)** as a purple solid, confirmed by ^1H NMR spectroscopy **(SI Figure S3).**

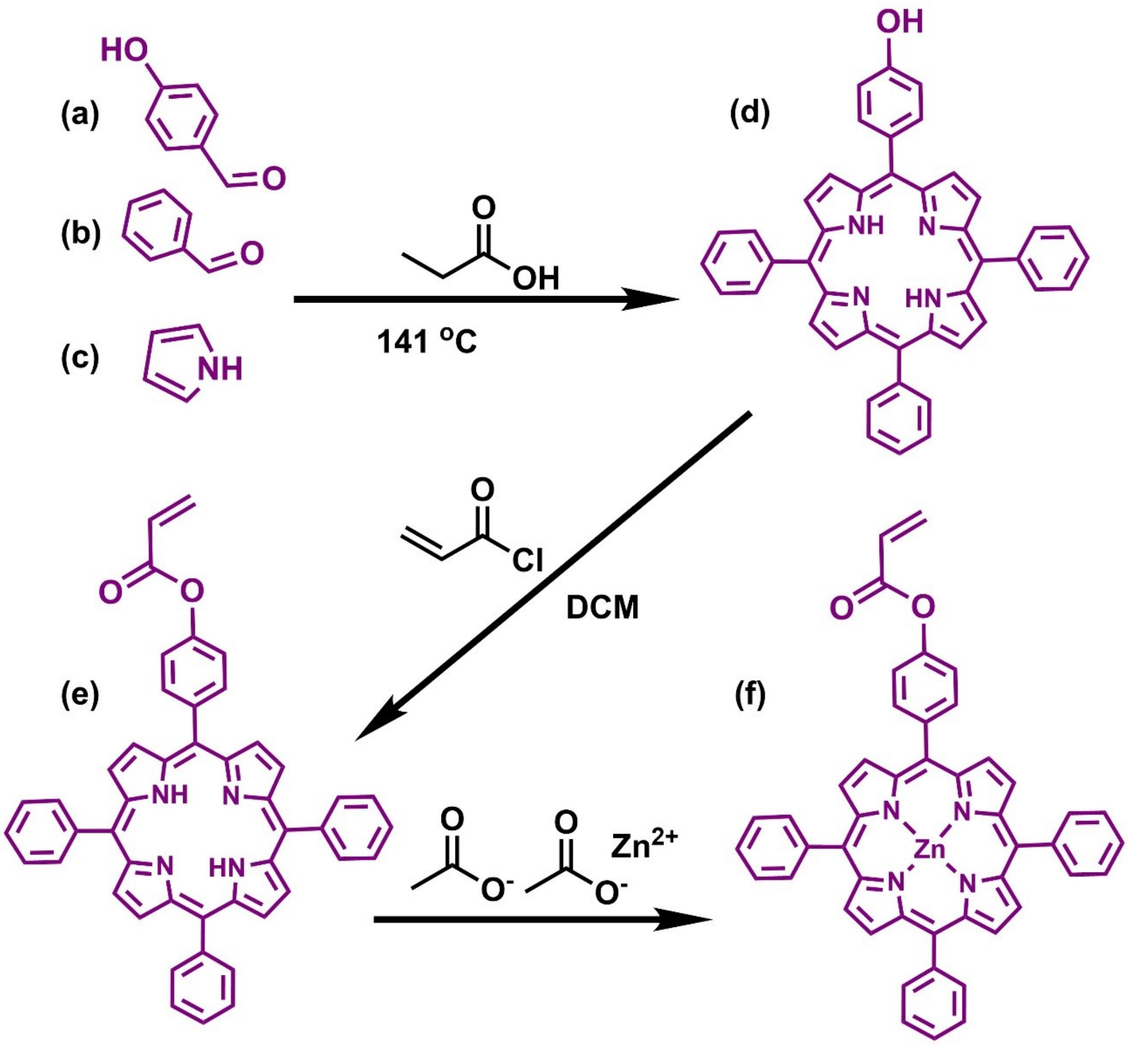

Scheme S2 The synthesis routes of vinyl functionalized photosensitizer acryl-ZnTPP

Polymer synthesis

ZnTPP-activated PET-RAFT polymerization

Homo-polymer P(C) and peptide mimicking polymer P(ABC) were synthesized using standard photoinduced energy/electron transfer-reversible addition-fragmentation chain transfer (PET-RAFT) polymerization (scheme S3). Briefly, monomer stock solutions containing 33 wt.% in DMSO and 5,10,15,20-tetraphenyl-21H,23H-porphine zinc (ZnTPP) dissolved in DMSO at a concentration of 1 mg mL^{-1} were prepared. The monomer solutions were added to a 2 mL vial along with BTPA, in amounts corresponding to a degree of polymerization (X_n) of 20. The ZnTPP photocatalyst was then added at a concentration of 100 ppm relative to the total monomer concentration in the solution. The vial was sealed with a rubber septum and deoxygenated headspace with N_2 for 10 min and then placed under a green LED light (λ_{max} = 530 nm, I_0 = 1.75 mW cm^2)

for 16 h at room temperature. The resulting P(C) and P(ABC) copolymer was further characterized by Size-exclusion chromatography (SEC), and ^1H NMR spectroscopy

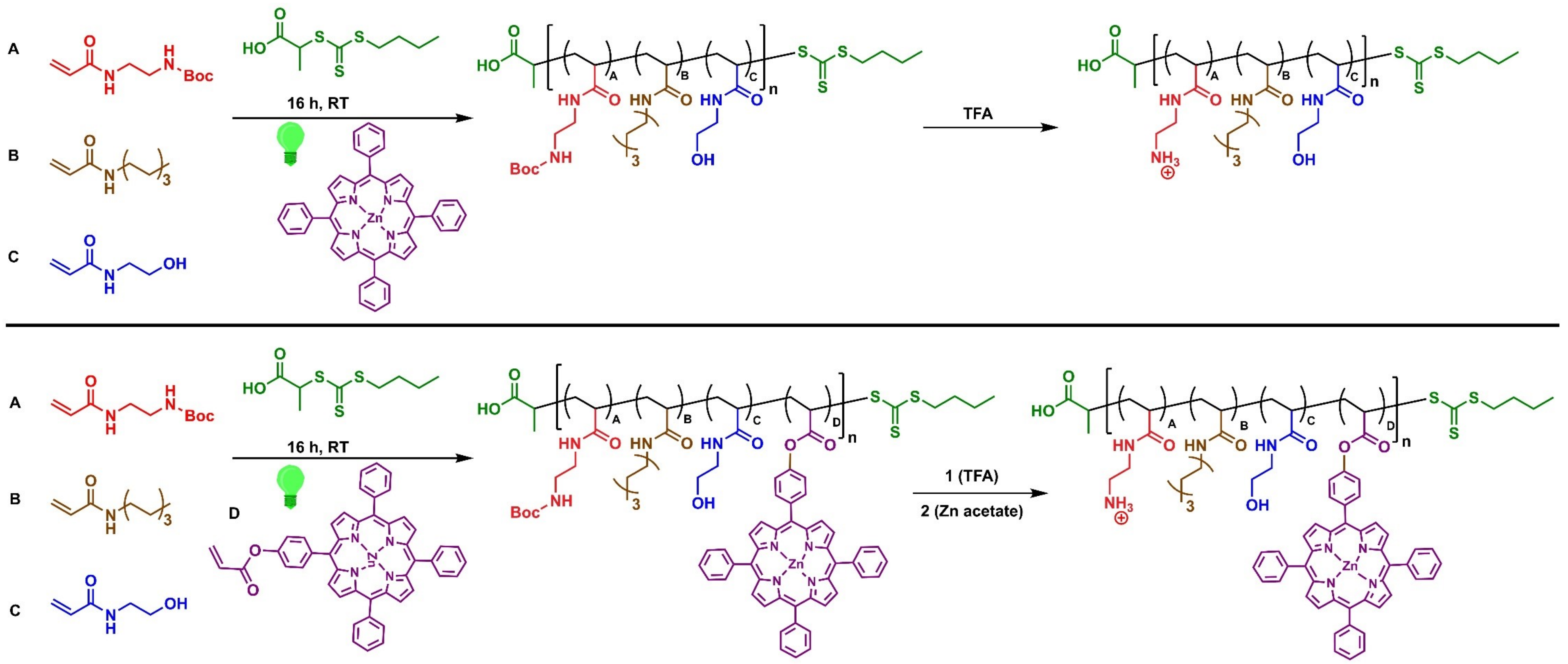

Scheme S3 Workflow for PET RAFT polymerization and post-polymerization Boc deprotection of P(ABC) (top) and P (ABCD) (bottom).

Note: Boc-AEm (A, red), HepAm (B, brown), HEAm (C, blue), and acryl-ZnTPP (D, purple).

Photoactivated polymers prepared by PET-RAFT polymerization

P(CD) and P(ABCD) were prepared using PET-RAFT polymerization, as previously reported, with slight modification **(Scheme S3)**.[2] Briefly, monomer stock solutions containing 33 wt.% in DMSO were added to a 2 mL vial along with BTPA, in an amount corresponding to $X_n \approx 20$. acryl-ZnTPP was added at a 1:1 molar ratio with BTPA, and the final solution was diluted to 10 wt.% in DMSO. The vial was sealed with a rubber septum and deoxygenated headspace with N2 for 10 min and then placed under a green LED light ($\lambda_{max} = 530$ nm, $I_0 = 1.75$ mW cm²) for 16 h at room temperature. The resulting photoactivated copolymer was further characterized using ^{1}H NMR spectroscopy, SEC, and UV-visible spectroscopy.

Post-polymerization:

Boc-deprotection using trifluoroacetic acid (TFA),

Following polymerization, samples containing the Boc-protected group (Boc-AEAm) underwent direct deprotection by adding trifluoroacetic acid (TFA) at a 20-fold molar excess relative to the Boc groups. The mixture was stirred at room temperature overnight, then precipitated with diethyl ether. After the reaction, the resulting polymer solutions were precipitated into ice-cold diethyl ether and centrifuged at 9000 rpm for 5 min. The final purified deprotected polymers were isolated, dried under vacuum, and analyzed using ^{1}H NMR spectroscopy and dynamic light scattering (DLS) **(SI, Figure S9).**

Photoactive polymer Re-metallization.

To compensate for potential zinc loss during polymer synthesis, polymers containing acryl-ZnTPP were re-metallized (**SI, Figure S10)**. The polymer was first dissolved in methanol, followed by the addition of zinc acetate dihydrate at a 10-fold molar excess relative to the porphyrin content. The mixture was stirred overnight at room temperature to ensure complete re-metallization. After reaction, methanol was evaporated, and the residue was redissolved in acetone while precipitating excess zinc salts. The acetone solution was then concentrated under reduced pressure, and the re-metallized polymer was precipitated by dropwise addition into ice-diethyl ether, centrifuged at 9000 rpm for 5 min, and dried under vacuum. Finally, all the polymers were further purified by dialysis using a 1 kDa dialyzer (thigh-throughput dialysis tubing, Pur-A-Lyzer, Sigma Aldrich) against DMSO, followed by precipitation in cold diethyl ether. The final polymers were then dried in vacuo.

Characterizations

Nuclear magnetic resonance (NMR) spectra were recorded on a Bruker Avance III spectrometer equipped with a sampleXpress probe operating at 400 MHz, using deuterated DMSO as the solvent. ^{1}H NMR was employed to determine monomer purity, polymer composition, and monomer conversion. Samples were typically prepared at a concentration of 5-10 mg mL^{-1}. Data analysis was conducted using TopSpin 4.1.4 software (2022, Bruker Biospin GmbH).

Diffusion-order NMR spectroscopy (DOSY) experiment was performed on 400 MHz Bruker NEO NMR system fitted with a Prodigy BBFO cryoprobe, using DMSO-d_6 as the solvent. Polymer P(ABCD) samples were typically prepared at a concentration of 5-10 mg mL^{-1}. A pulse-field gradient stimulated echo sequence with bipolar gradient (BPP-LED) was used for diffusion measurement at 298 K. Data processing and diffusion coefficient extraction were carried out using MestReNova 14.2.1-27684.

Size-exclusion chromatography (SEC) for molecular weight distributions and dispersity, and UV detection of the polymer and acryl-ZnTPP integration, were analyzed using a Shimadzu liquid chromatography system equipped with a Shimadzu refractive index detector and three MIX C columns. The analysis was performed at 50 °C with a flow rate of 1 mL/min. DMAc was used as the eluent, containing 0.3 g/L LiBr and 0.5 g/L 2,6-di-butyl-4-methylphenol. Calibration was performed using narrowly distributed poly(methyl methacrylate) (PMMA) standards with molecular weights ranging from 200 to 10^6 g/mol. For analysis, polymers were diluted in DMAc, filtered through a 0.45 µm filter, and then injected into the column for measurement.

Dynamic light scattering (DLS) analysis was performed on a Malvern Zeta Sizer Nano ZS apparatus equipped with a He-Ne laser operated at λ = 633 nm and at a scattering angle of 173°. Samples were analyzed in a folded capillary cell (DTS1070) at 25 °C for 60 s in equilibrated conditions. The polymer concentration was adjusted to 1.5 mg mL-1 in Milli-Q water, and the solution was filtered through a 0.45 µm syringe filter before analysis. Each measurement was performed in triplicate.

UV-visible light spectra were acquired using a Varian Cary 300 spectrophotometer. Measurement was conducted in a 1 cm path-length glass cuvette, with the instrument zeroed at 800 nm using a solvent (methanol

or water, depending on the sample). Baseline correction was applied over the 200-800 nm range to ensure accurate absorbance profiles of the polymer and control solution in the solvent.

Biological experiment:

Media Preparation:

Phosphate-buffered saline (PBS, pH 7.4) was prepared by dissolving one Oxoid™ phosphate-buffered saline tablet in 100 mL of Milli-Q water, then autoclaving. **Modified Roswell Park Memorial Institute medium (RPMI-1640)** medium was prepared by dissolving 18 g of D-glucose, 34.53 g of MOPS and 10.4 g of RPMI-1640 powder (containing L-glutamine and phenol red, without bicarbonate) in 980 mL of Milli-Q water, adjusting the pH to 4.0 using HCl and NaOH, and bringing the to 1 L before filter sterilization and storge at 4 °C. **Sabouraud Dextrose Agar (SDA)** was prepared by autoclaving 8 g of mycological peptone and 16 g of technical agar in 720 mL of milli-Q water, then adding 80 mL of filter-sterilized 40% (w/v) D-glucose before pouring plates. **The yeast extract peptone dextrose (YEPD)** agar was prepared by autoclaving a solution of 8 g yeast extract, 16 g technical agar, and 16 g mycological peptone in 760 mL of Milli-Q water, then adding 40 mL of filter-sterilized 40% (w/v) D-glucose solution.

Fungal Strain and Culture Condition:

The *Candida* strains used in this study include the *Candida albicans* reference strain *(*SC5314[3]), a clinical isolate of *C. albicans* (b30708/5[4]) and a type strain of *Candida parapsilosis (*ATCC 22019[5]). The strains were revived from glycerol stocks by streaking onto YEPD agar or SDA plates and incubating for 48 h at 30 °C. For long-term storage, stocks were prepared from an overnight YEPD broth culture and stored at -80 °C in 50% (v/v) sterile glycerol. For the MIC assay, the strain was revived from cultures on YEPD agar or SDA plates and then stored at 4 °C for up to 2 weeks.

Minimum Inhibitory Concentration (MIC) Assay:

Minimum inhibitory concentration (MIC) values were determined using a broth microdilution assay according to the Clinical and Laboratory Standards Institute (CLSI) protocol, with minor modifications.[6] Briefly, stock solutions of the polymers (5 mg mL^{-1}) were prepared in Milli-Q water. Amphotericin B (1 mg mL^{-1}) and fluconazole (1 mg mL^{-1}) were dissolved in 50% (v/v) Milli-Q water/DMSO. *Candida* species were cultured on SDA plates for 48 h at 30 °C, and subcultures were prepared in sterile Milli-Q water. The polymer stock

solution was diluted to 1024 μg mL^{-1} in RPMI-1640 media and then serially twofold diluted in flat-bottom 96-well, non-pyrogenic, polyester microplates to yield final test concentrations ranging from 512 μg mL^{-1} to 2 μg mL^{-1}. A single colony of *Candida* species was suspended in 1 mL of sterile Milli-Q water, counted using a hemocytometer, and adjusted to 5×10^6 cells mL^{-1}. This suspension was further diluted to 1:1000 in RPMI-1640. Doubling dilutions of the polymers and antifungal drugs (100 μL) were mixed with an equal volume of *Candida* cell suspension in a 96-well plate, yielding a final inoculum of 2.5×10^3 cells per well, control wells containing only media (negative control) and untreated *Candida* cells (positive control). The plates were incubated at 35 °C for 24 h, and growth was measured at 600 nm using a microtiter plate reader (SpectraMAX 190, Molecular Devices). MIC was defined as the lowest antifungal concentration that inhibited >90% of cell growth relative to the untreated control. Each assay included two technical replicates repeated in at least a biological triplicate. The percentage of fungal cell inhibition was calculated using:

$$Percentage\ Inhibition = \left(\frac{OD_{Positive} - OD_{polmer}}{OD_{Positive} - OD_{negative}}\right) \times 100$$

where OD $_{polymer}$ is the absorbance measured at 405 nm for the MIC plate incubated at 35 °C for 24 h, OD $_{positive}$ is the absorbance of the positive control, and OD $_{negative}$ is the absorbance of the negative control.

Minimum fungicidal concentration (MFC):

Following the determination of the MIC, 100 μL of aliquots were collected from wells showing no visible fungal growth at a concentration equal to or greater than the MIC.[7] Each aliquot was plated onto SDA plates. The plates were incubated at 30 °C for 48 h. The MFC was defined as the lowest concentration of polymers or antifungal agents that prevented visible fungal colony formation on the SDA plate, indicating complete fungicidal activity.

Hemolytic Assay:

The hemolytic activity of the polymers against fresh defibrinated sheep blood (from Serum Australia) was assessed as described previously.[8] Briefly, fresh defibrinated sheep blood was diluted 1:20 in PBS (pH 7.4), and red blood cells (RBCs) were pelleted by centrifugation (1000 x *g*) for 10 min, followed by three washes with PBS. The washed RBCs were then resuspended to a final concentration 5% (v/v) in PBS. Serial dilutions

of polymers (150 μL) at concentrations ranging from 2000 μg mL^{-1} to 250 μg mL^{-1} and antifungal agents (fluconazole and amphotericin) at concentrations ranging from 125 μg mL^{-1} to 7.8 μg mL^{-1} were prepared in sterilized centrifuge tubes (1.5 mL), followed by the addition of 150 μL of RBC suspension. PBS buffer was used as a negative control, and Triton-X 100 (1% (v/v) in PBS) was used as a positive hemolytic control. Preliminary absorbance readings of the polymer solution in PBS were recorded to account for any potential spectra overlap from photosensitizer-containing polymers with the hemoglobin absorbance peak. The test tubes were incubated at 37 °C while shaking at 150 rpm for 2 h. After incubation, the samples were centrifuged at room temperature (1000 x *g*, 8 min), and 100 μL of supernatant was transferred into a 96-well microplate. and read at 408 nm using a microtiter plate reader (FLUOstar Omega, BMG Labtech). All experiments were performed in triplicate. The percentage hemolysis was calculated using the following general equation:

$$\% \, Hemolysis = \frac{A_{Polymer} - A_{negative}}{A_{Positive} - A_{negative}} \times 100$$

Where $A_{polymer}$ is the absorbance of the polymer-treated supernatant, $A_{positive}$ is the absorbance of the positive control, and $A_{negative}$ is the absorbance of the negative control.

Optical setup and photochemical Assay

Light source photoreactor setup:

Photodynamic irradiation was performed using custom-designed red and green LED light panels integrated into a 96-well plate illumination system (Bio Research Center Co., Ltd). The device featured adjustable intensity settings and uniform light distribution across all wells (**Figure S12).** Green and red LEDs (Green, $\lambda \approx 530$ nm; Red, $\lambda \approx 630$ nm) were used to activate acryl-ZnTPP-containing polymers during photodynamic assays. The light intensity was maintained at a consistent 13V for the red and green sources, and the green-light intensity was measured at 3.45 mW/cm^2 at the surface of the assay where the 96-well plate *Candida* cells were cultured. Exposure times were standardized to 60 min across all experiments unless otherwise specified.

Singlet Oxygen Quantification Using DMA Quenching

The singlet oxygen ($^1O_2$) generation capacity of the polymers P(ABCD) was evaluated using 9,10-dimethylanthracene (DMA) as a selective $^1O_2$ probe (**Scheme S4**). Polymer samples were prepared at a

concentration of 50 µg mL^{-1} in Dimethylformamide (DMF), and DMA was added at a 1:1 molar ratio relative to the calculated acryl-ZnTPP content in the polymer. The resulting solutions were transferred to quartz cuvettes and irradiated with a green LED light source (λ max = 530 nm, 3.45 mW cm^2). The decrease in DMA absorbance, corresponding to its photochemical conversion to endoperoxide, was monitored at 380 nm at defined time intervals (0, 5, 15, 20, 25, and 30 min) using a UV-Vis spectrophotometer**.** A calibration curve was constructed for DMA, confirming linearity according to the Beer-Lambert law up to 1 mM.

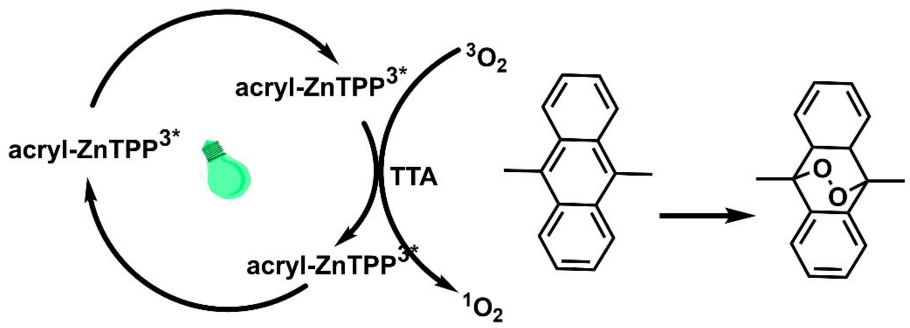

Scheme S4 The photochemical reaction of 9,10-dimethylanthracene (DMA) consistently occurs in the presence of acryl-ZnTPP.

Note: Upon green light irradiation, the polymer containing acryl-ZnTPP generates $^1O_2$ via a type II photosensitization pathway mediated by triplet-triplet energy transfer (TTA). The reactive $^1O_2$ subsequently undergoes a [4+2] cycloaddition with the anthracene moiety of DMA, forming the corresponding endoperoxide adduct.

Reactive Oxygen Species Scavenger Assay

To investigate the types of Reactive Oxygen Species (ROS) involved in the antifungal mechanism, Scavenger inhibition studies were conducted using selective chemical quenchers.[9] The following scavengers were employed at a final concentration of 25 mM each: DMSO, a superoxide radical scavenger; Ascorbic acid (AscA), a single oxygen scavenger; Mannitol hydroxyl, a radical scavenger; and N-acetylcysteine (NAC), a hydroperoxyl radical scavenger. The antifungal activity of each polymer was evaluated in the presence and absence of scavengers using the broth microdilution method, as described in the MIC assay following the CLSI guidelines.[6] Test conditions included incubation of *Candida* species with polymer samples under dark

and light irradiation, in the presence of the corresponding scavengers. Changes in MICs were recorded to determine the contribution of specific ROS to fungal inactivation. The control assay confirmed that the scavengers themselves exhibited no antifungal activity at the tested concentration or at dilutions above those used in the experiments **(Table S1)**.

Supporting information - Figures and Tables

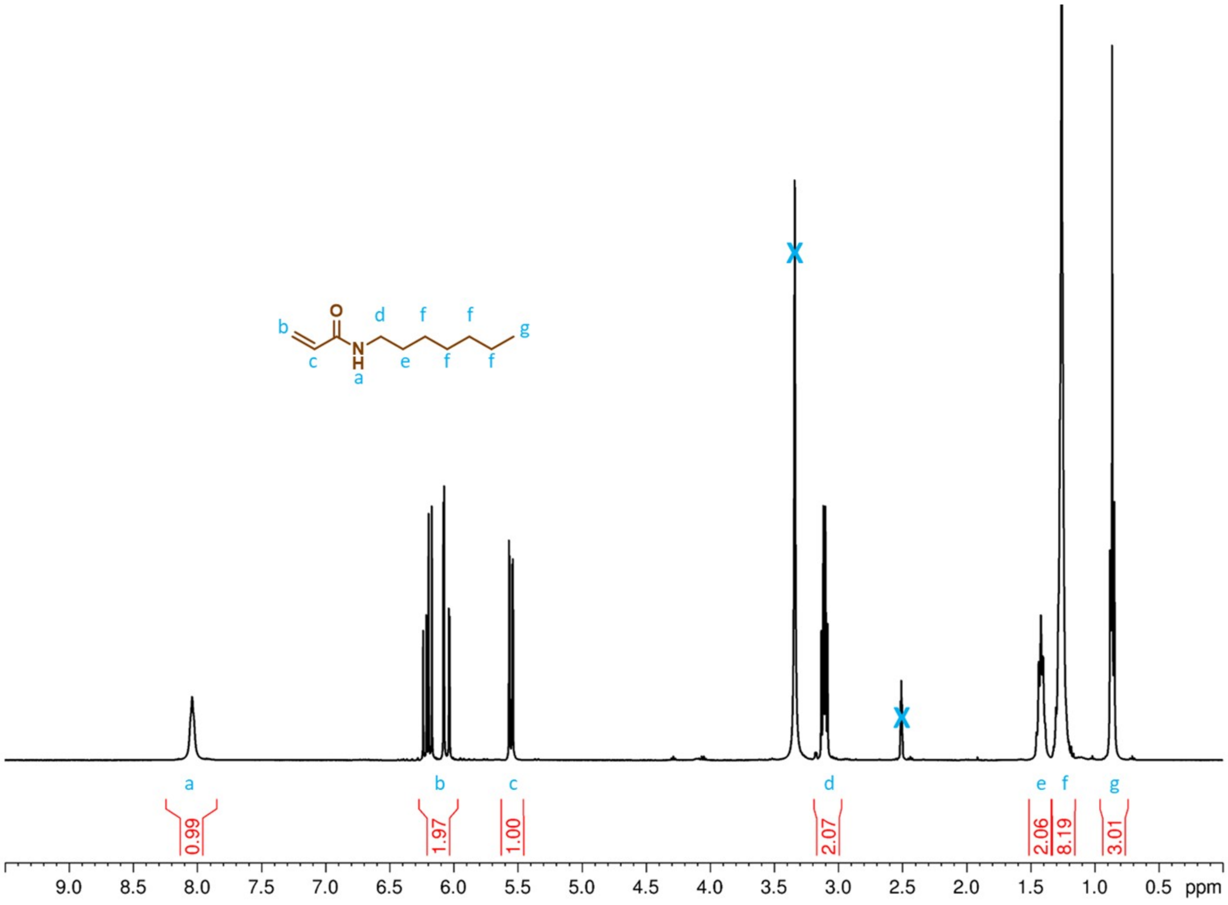

Figure S1. ^{1}H NMR spectrum of HepAm in DMSO-d6. Labels a-g correspond to the attributes of the protons of the monomer.

Note: For all figures, the blue crosses in the ^{1}H NMR spectra indicate signals attributed to solvent residues. Alphabet Labels (e.g., a-g in Figure S1) correspond to the proton assignments of the monomers or polymer structures, and the integration values are shown in red.

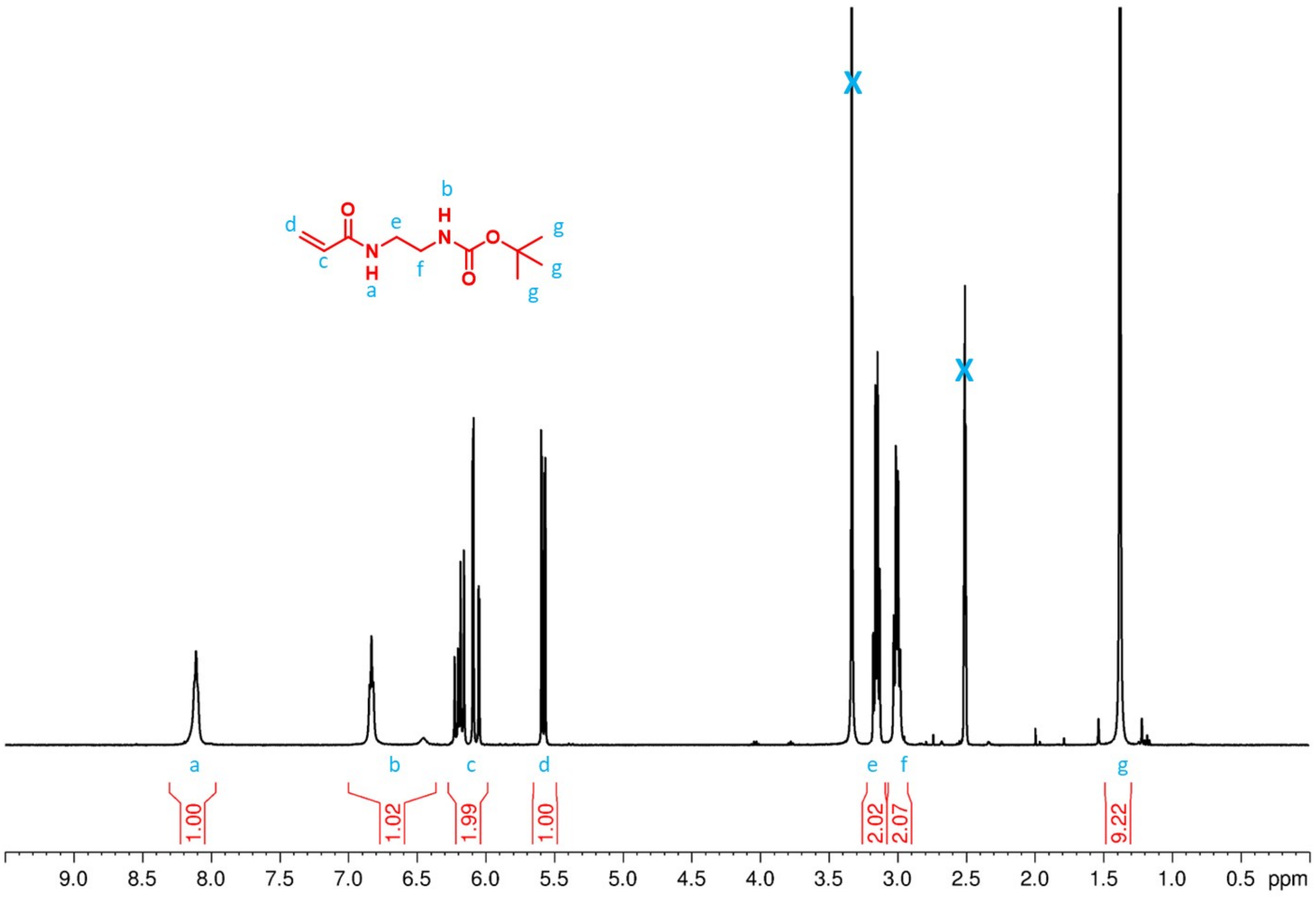

Figure S2. ^{1}H NMR spectrum of Boc-AEAm in DMSO-d6. Labels a-g correspond to the attributes of the protons of the monomer.

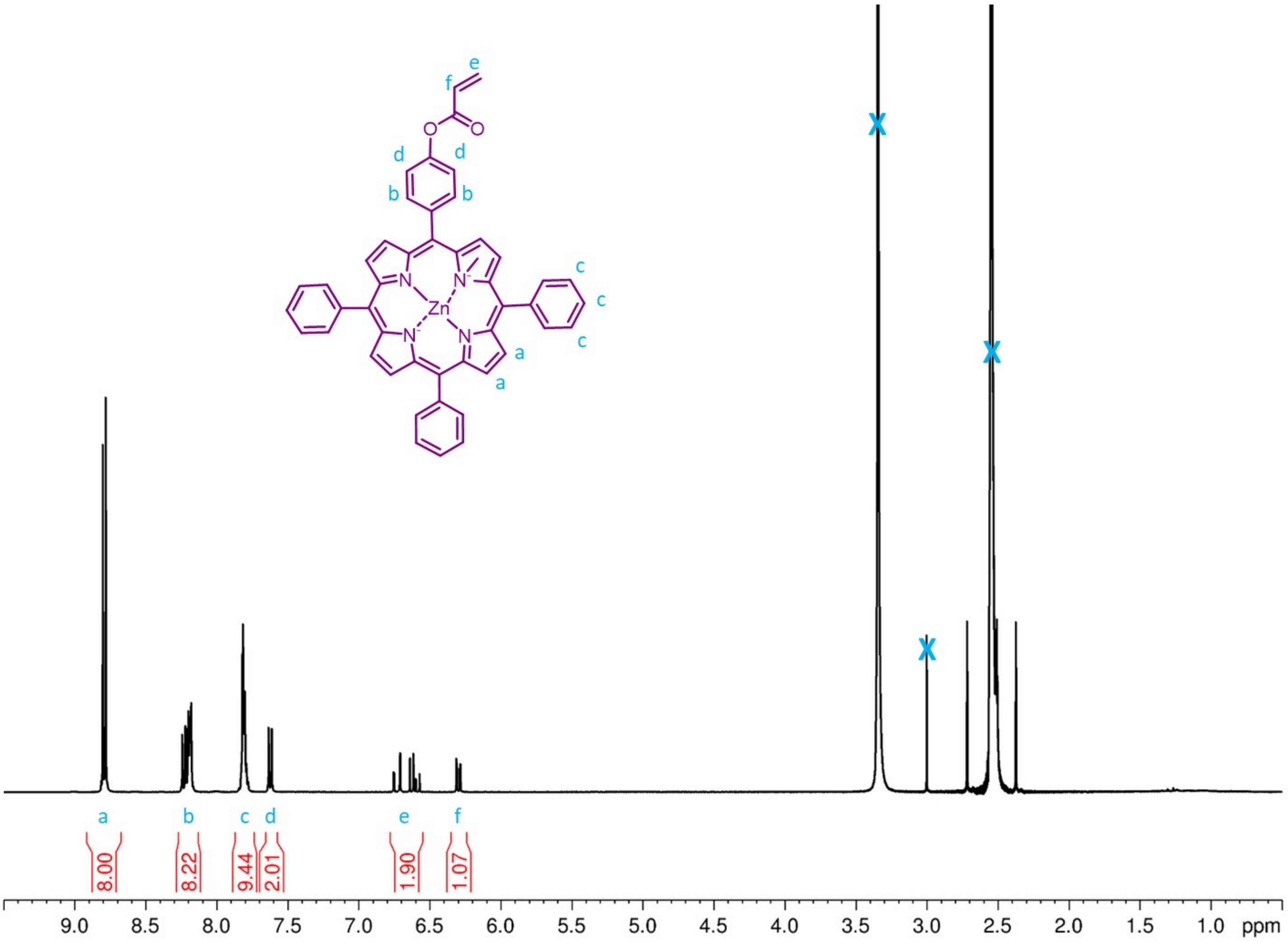

Figure S3. ^{1}H NMR spectrum of acryl-ZnTPP in DMSO-d6.

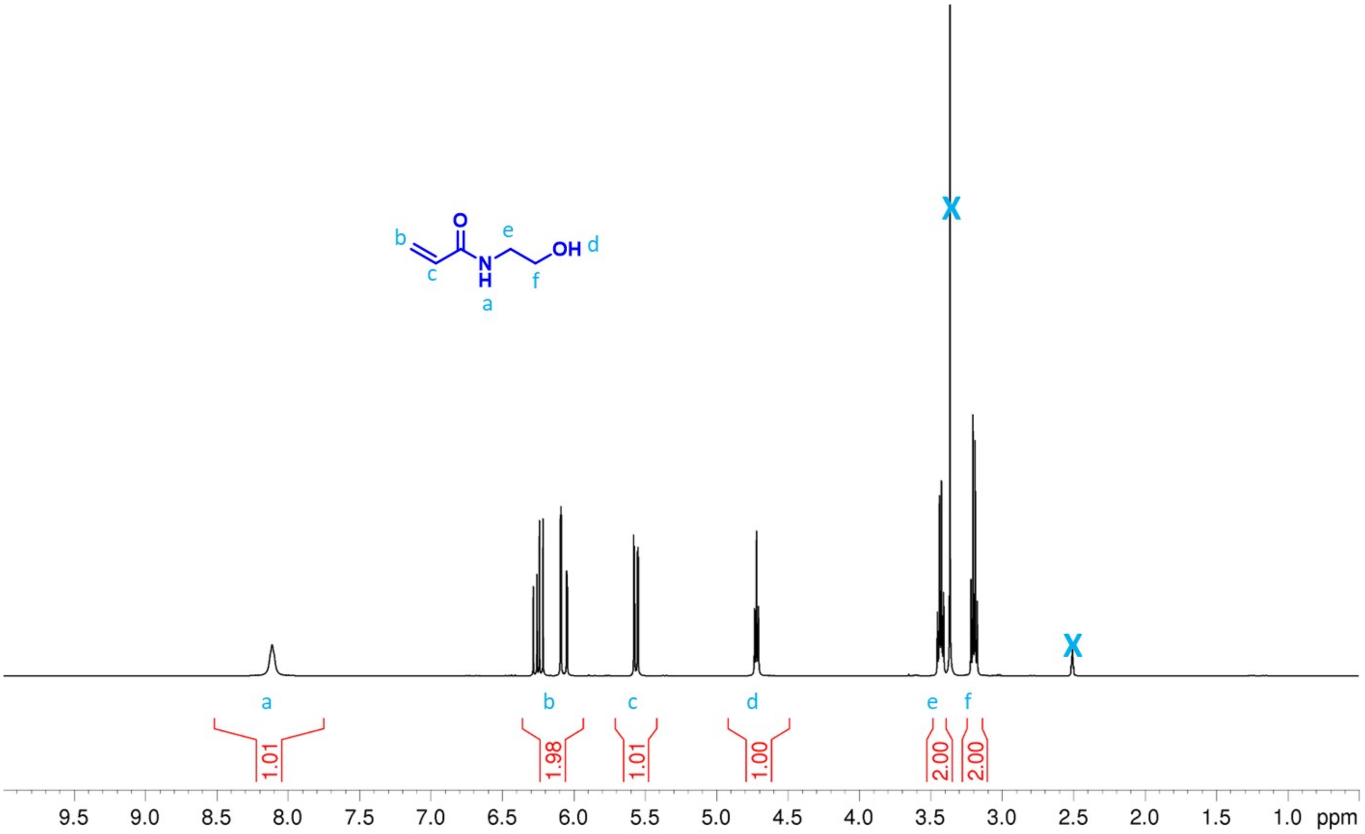

Figure S4. ^{1}H NMR spectrum of HEAm in DMSO-d6.

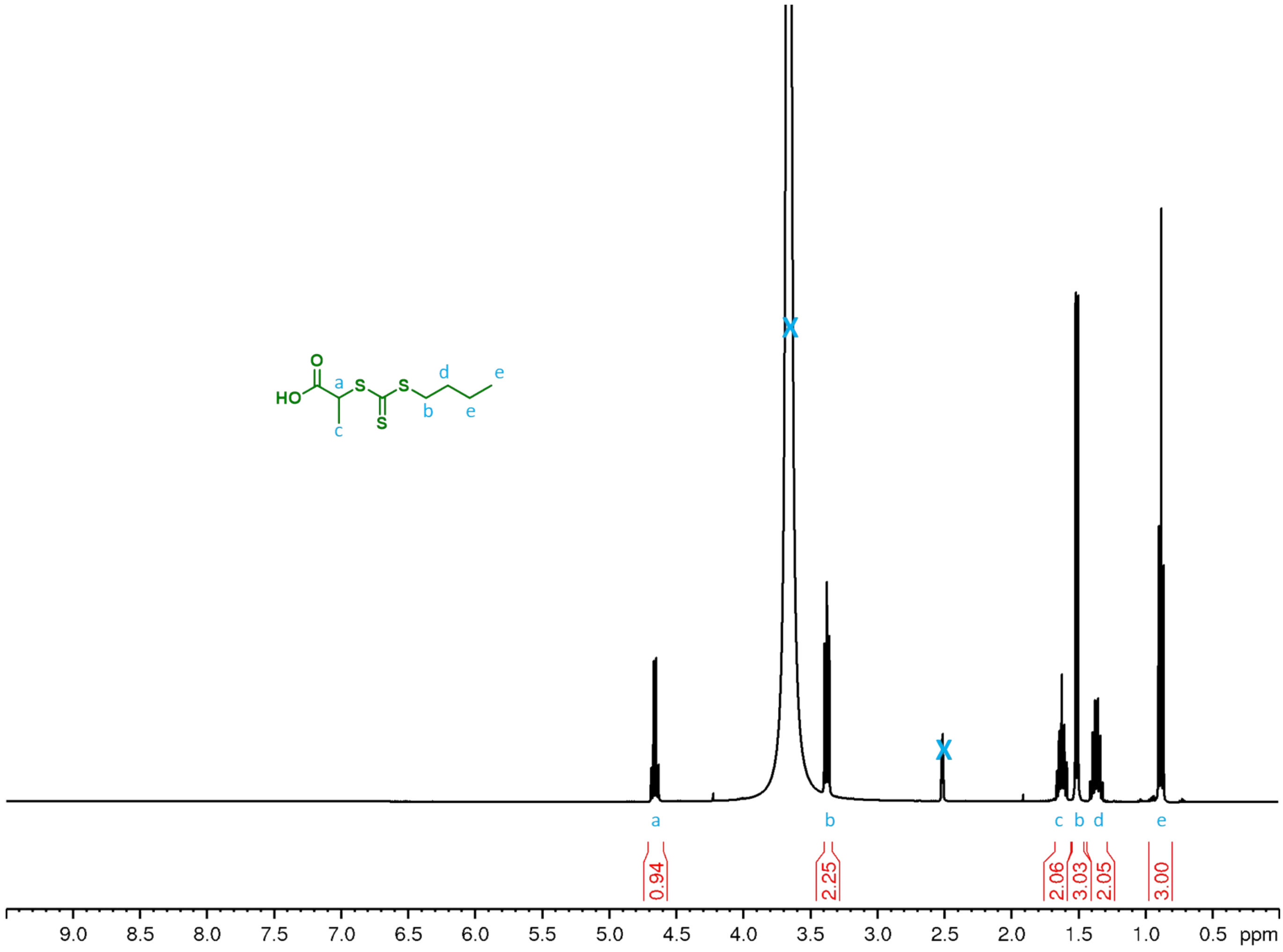

Figure S5. ^{1}H NMR spectrum of BTPA in DMSO-d6.

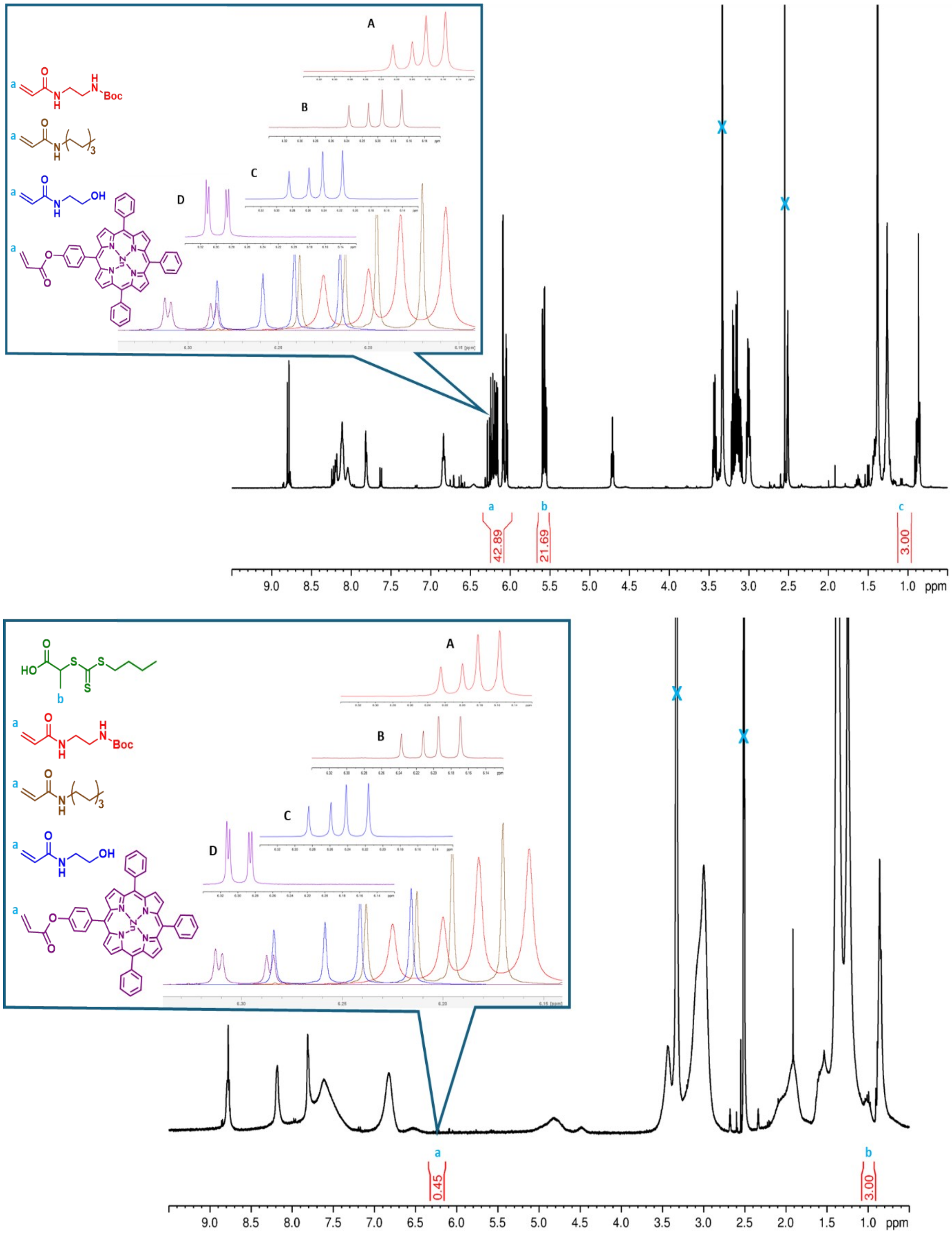

Figure S6. ^{1}H NMR spectrum of the reaction mixture used for the preparation of P(ABCD) before polymerization (top) and ^{1}H NMR spectrum of P(ABCD) after polymerization (bottom) in DMSO-d_6. Labels a and b correspond to the integration peaks of the proton monomers in the polymer structure—signal "b" used as a reference for the BTPA integral observed in **Figure S5**.

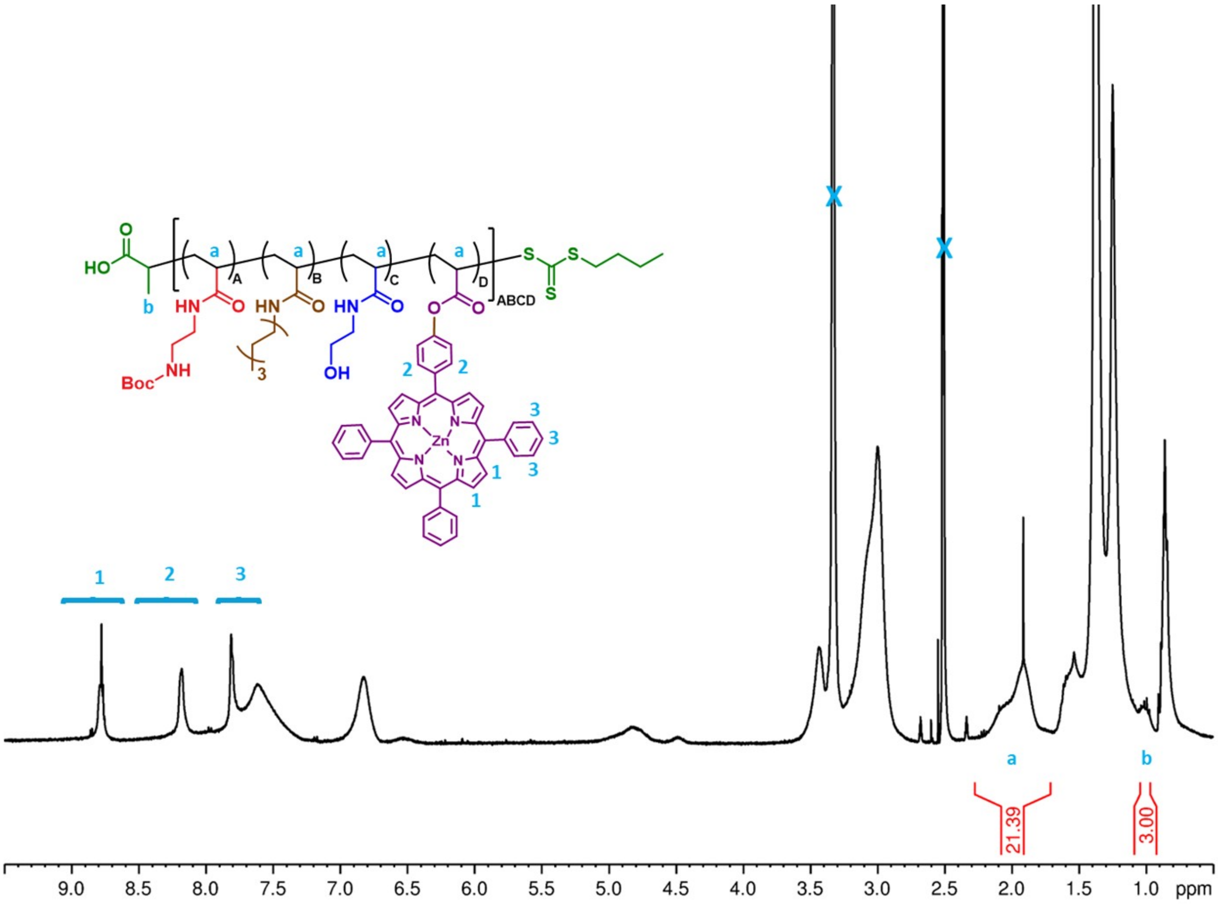

Figure S7. ^{1}H NMR spectrum of Boc-protected P(ABCD) before purification in DMSO-d_6. Integral region a and b correspond to the proton signals of the polymer CH backbone after complete polymerisation and BTPA integral, respectively. Labels 1–3 indicate distinct peaks assigned to acryl-ZnTPP after polymerization.(**see Figure S3)**

Note: The degree of polymerization (X_n) is calculated based on the polymer CH backbone and the integration of the CH_3 (BTPA, b) signal

$$X_n \approx \int a \approx 21.4$$

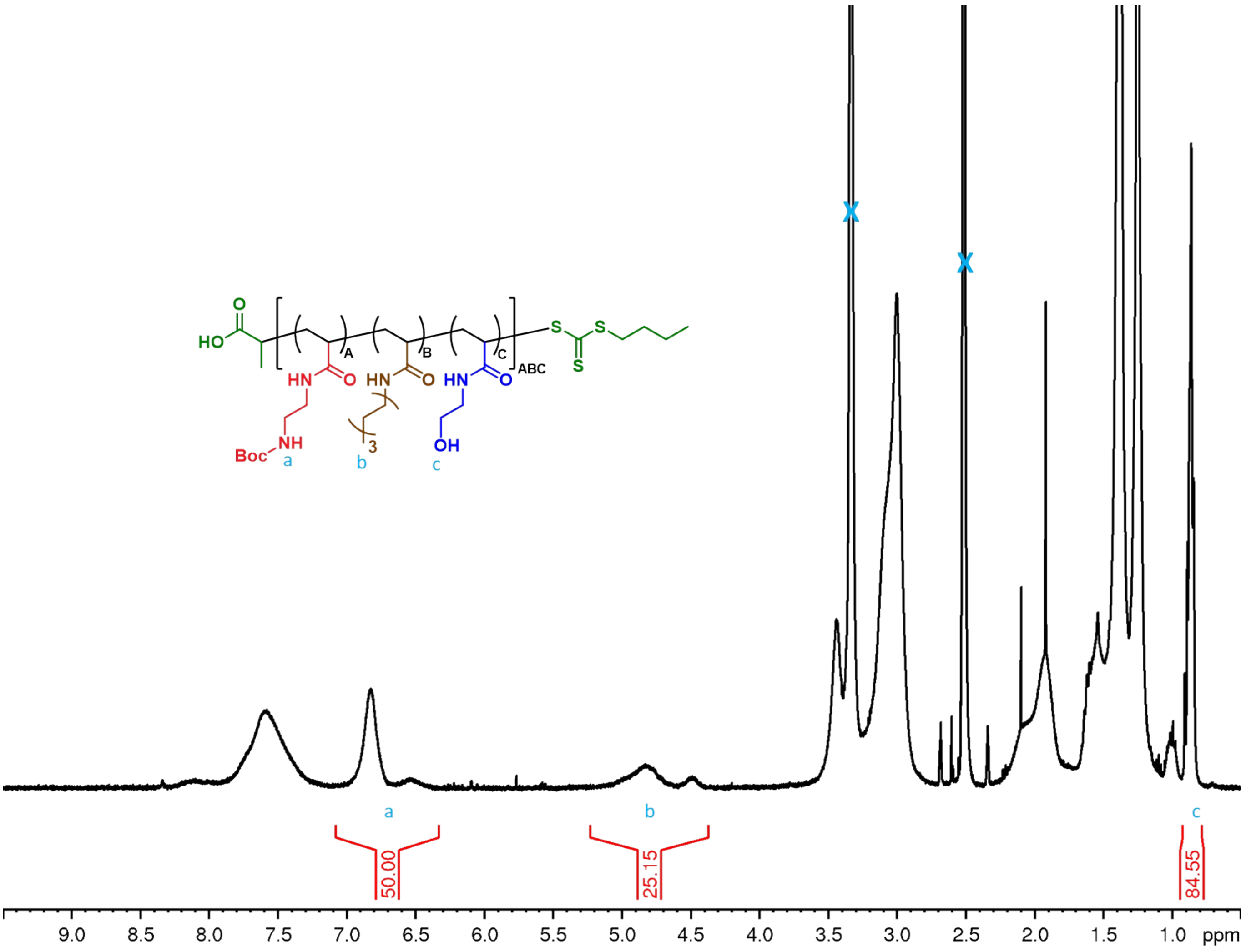

Figure S8. ^{1}H NMR spectrum of **P(ABC)** before deprotection in DMSO-d_6. Labels a–c correspond to the integration peaks of the proton monomers in the polymer structure. label c corresponds to the CH_3 of the HepAm signal, which overlaps with the signal of BTPA.

Composition and molecular weight calculation:

$$\%\ \text{Boc-AEAm} = \left(\frac{\int a}{\int 1 + \frac{(\int c - 3)}{3} + (\int b - 1)}\right) x100$$

$$\%\ \text{Boc-AEAm} = \left(\frac{50}{50 + \frac{(84.55 - 3)}{3} + (25.17 - 1)}\right) x100$$

% Boc-AEAm = 50 %

$$\%\ \text{HepAm} = \left(\frac{\frac{(\int c - 3)}{3}}{\int 1 + \frac{(\int c - 3)}{3} + (\int b - 1)}\right) x100$$

$$\%\ \text{HepAm} = \left(\frac{\frac{(84.55 - 3)}{3}}{50 + \frac{(84.55 - 3)}{3} + (25.15 - 1}\right) x100$$

% HepAm = 26 %

$$\%\ \text{HEAm} = \left(\frac{(\int b - 1)}{\int 1 + \frac{(\int c - 3)}{3} + (\int b - 1)}\right) x100$$

$$\%\ \text{HEAm} = \left(\frac{(25.15 - 1)}{50 + \frac{(84.55 - 3)}{3} + (25.15 - 1)}\right) x100$$

% HEAm = 24 %

Molecular weight (M_n) of **P(ABC)**= X_n **(**($M_{nA} \times F_A$) + ($M_{nB} \times F_B$) + ($M_{nC} \times F_C$)**)** + $M_{n\ RAFT}$ +

M_n of **P (ABC)**= 20 **(**(214.3 × 0.50) + (169.3 × 0.25) + (115 × 0.25)**)** + 238.38

M_n of **P(ABC)**= 3811 g/mol, =3.8 kg/mol

Note: the low vinyl signals at δ 5.5−6.3 ppm indicate almost complete monomer conversion (>98%). Label 4 corresponds to an integral proton positioned adjacent to the last monomer in the polymer structure. The molecular weights of the monomers $M_{n\mathbf{A}}$ (Boc-AEAm, 214.3 g/mol), $M_{n\mathbf{B}}$ (HepAm, 169.3 g/mol*),* $M_{n\mathbf{C}}$ *(*HEAm*,* 115 g/mol*),* and M_{nRAFT} (BTPA, 238.38 g/mol) were determined using ChemDraw. Monomer ratio composition was converted to a fraction (*f*) from the percentage composition. For X_n, refer to Figure S12.

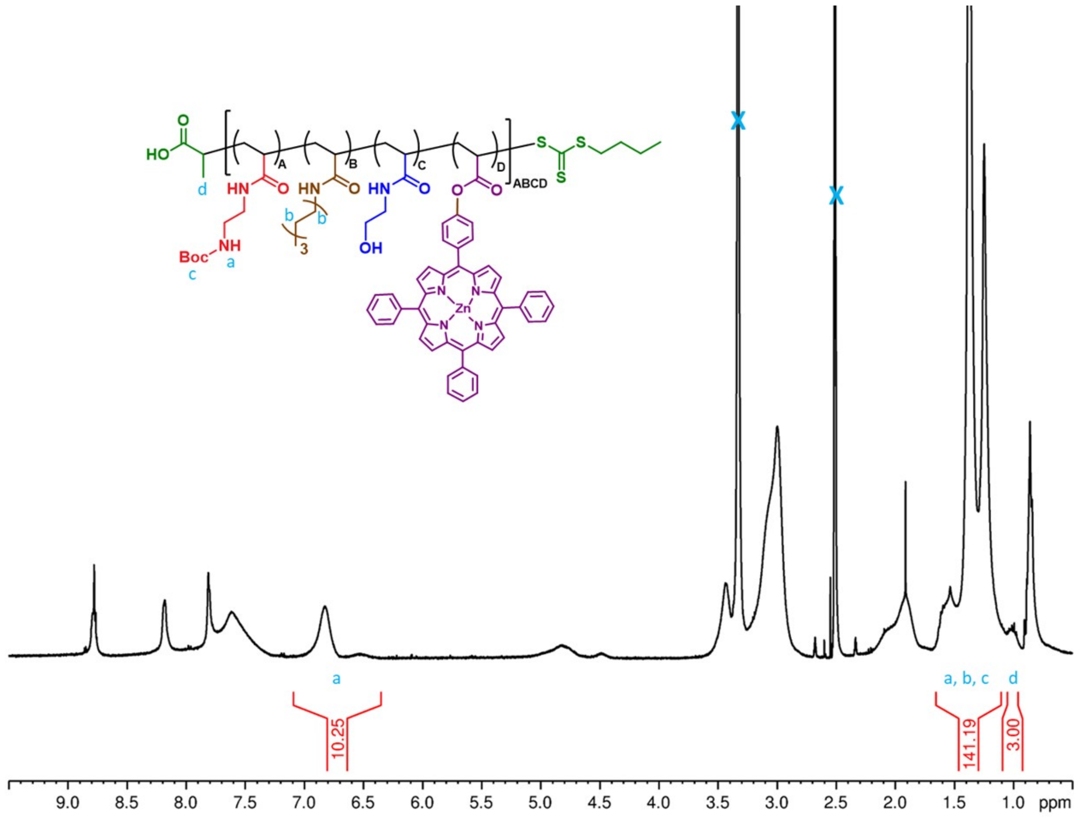

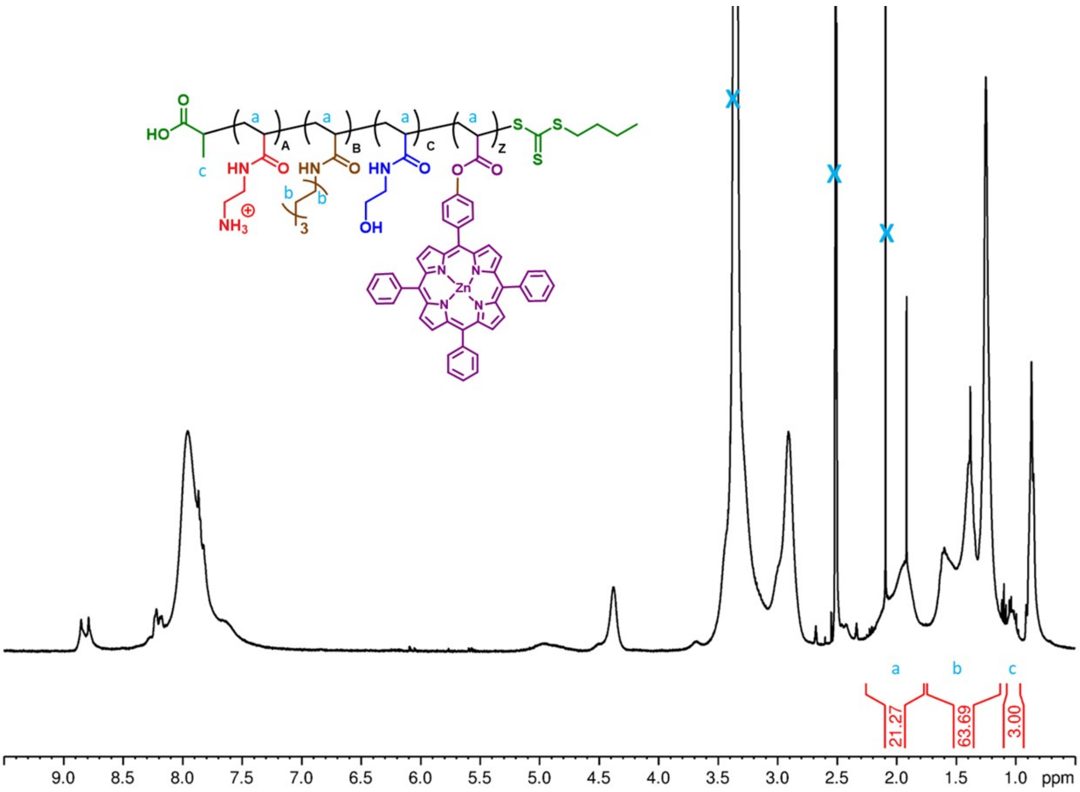

Figure S9. ^{1}H NMR spectrum of Boc-protected P(ABCD) (top) and ^{1}H NMR spectrum of P(ABCD) of Boc-removed (bottom) before purification in DMSO-d_6. Labels a -b correspond to the integration peaks of the proton signals in the polymer structure

Note: the absence of signals in the Boc-removed ^{1}H NMR spectrum (bottom) at 6.8 and 1.4 ppm confirms the successful Boc-group removal.

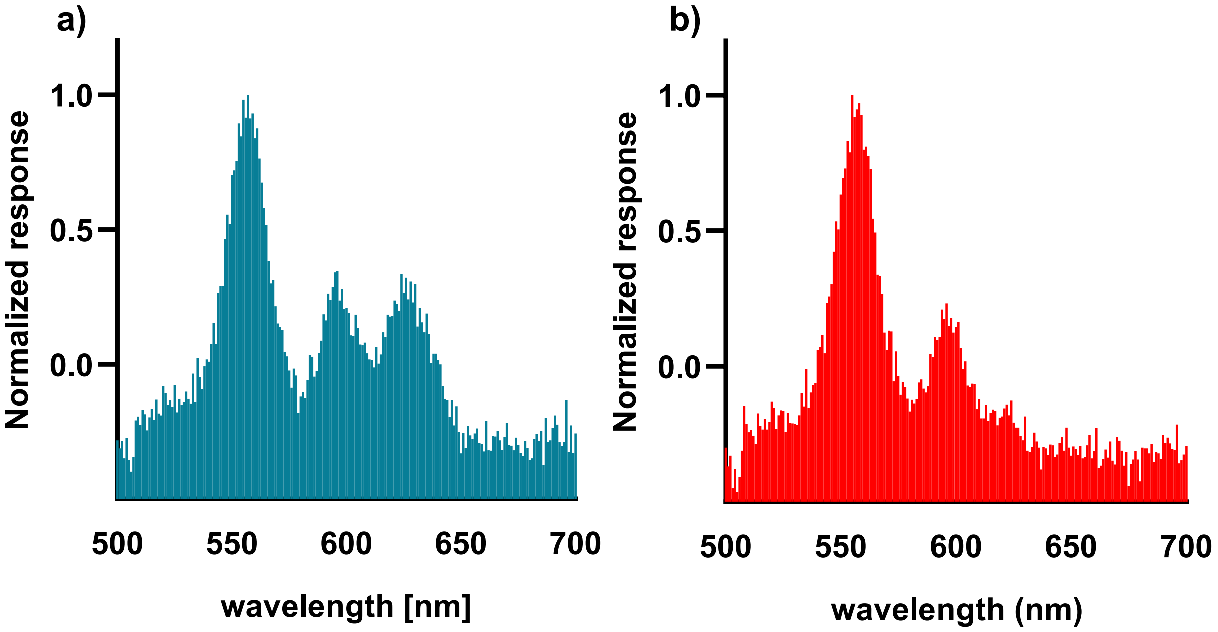

Figure S10. UV-visible light spectra of the P(ABCD) before (a) and after (b) Zn(II) metalation. This figure shows the absence of the band at ~590 nm and an increase in the band peak at ~550 nm upon Zinc coordination

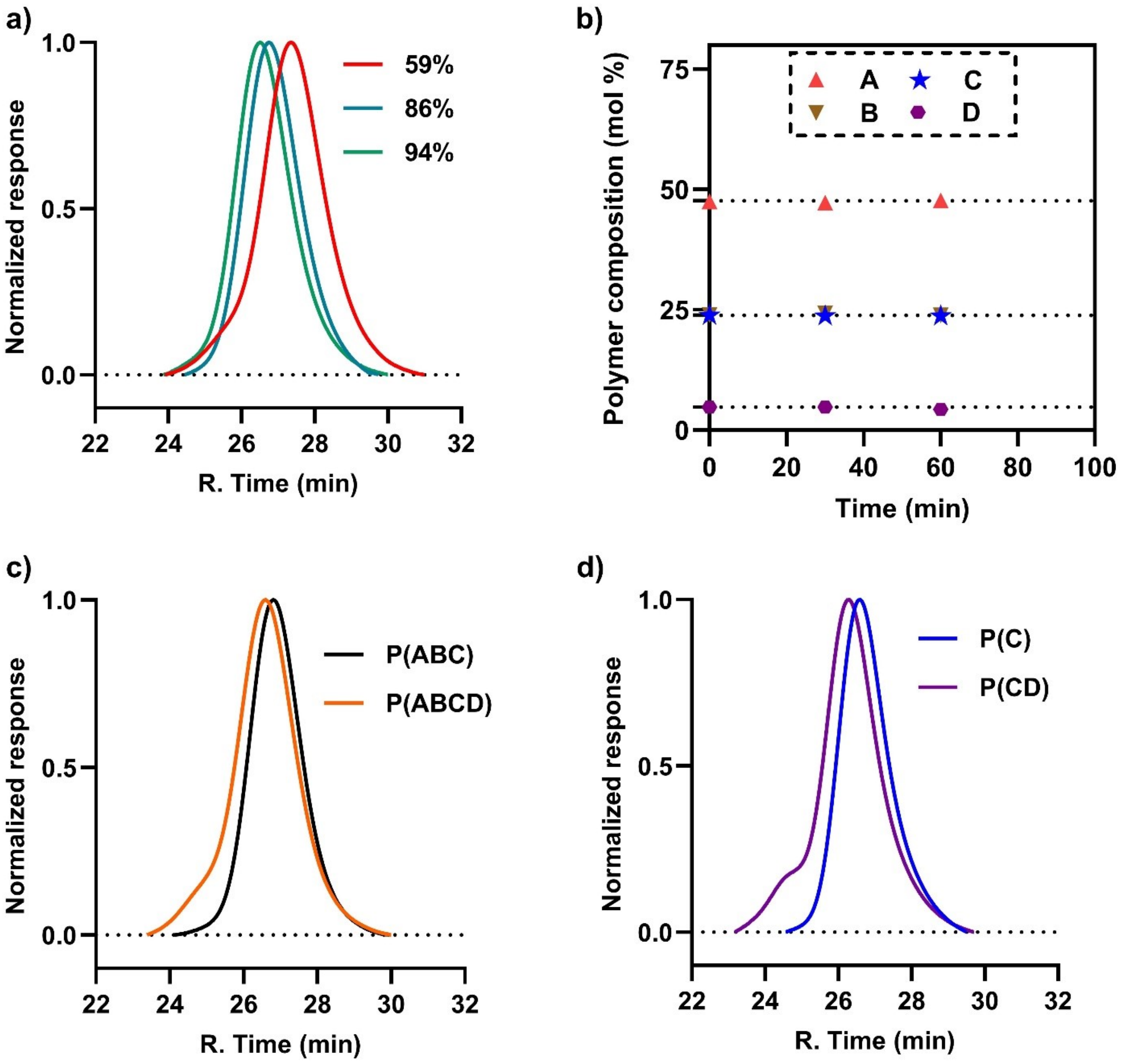

Figure S11. PET- RAFT polymerization kinetics and composition analysis. (a) Molecular weight distributions (MWD) of P(ABCD) polymer at increasing reaction time obtained by SEC analysis using DMAc as eluent and PMMA calibration standards (the indicated percentage corresponds to overall monomer conversion (mol%) determined by ^{1}H NMR analysis), (b) Comparison of MWD of P(ABC) and P(ABCD) obtained by SEC analysis, (c) Polymer composition (mol%) as a function of polymerization time, and (d) MWD for polymer P(C) and P(CD) obtained by SEC analysis.

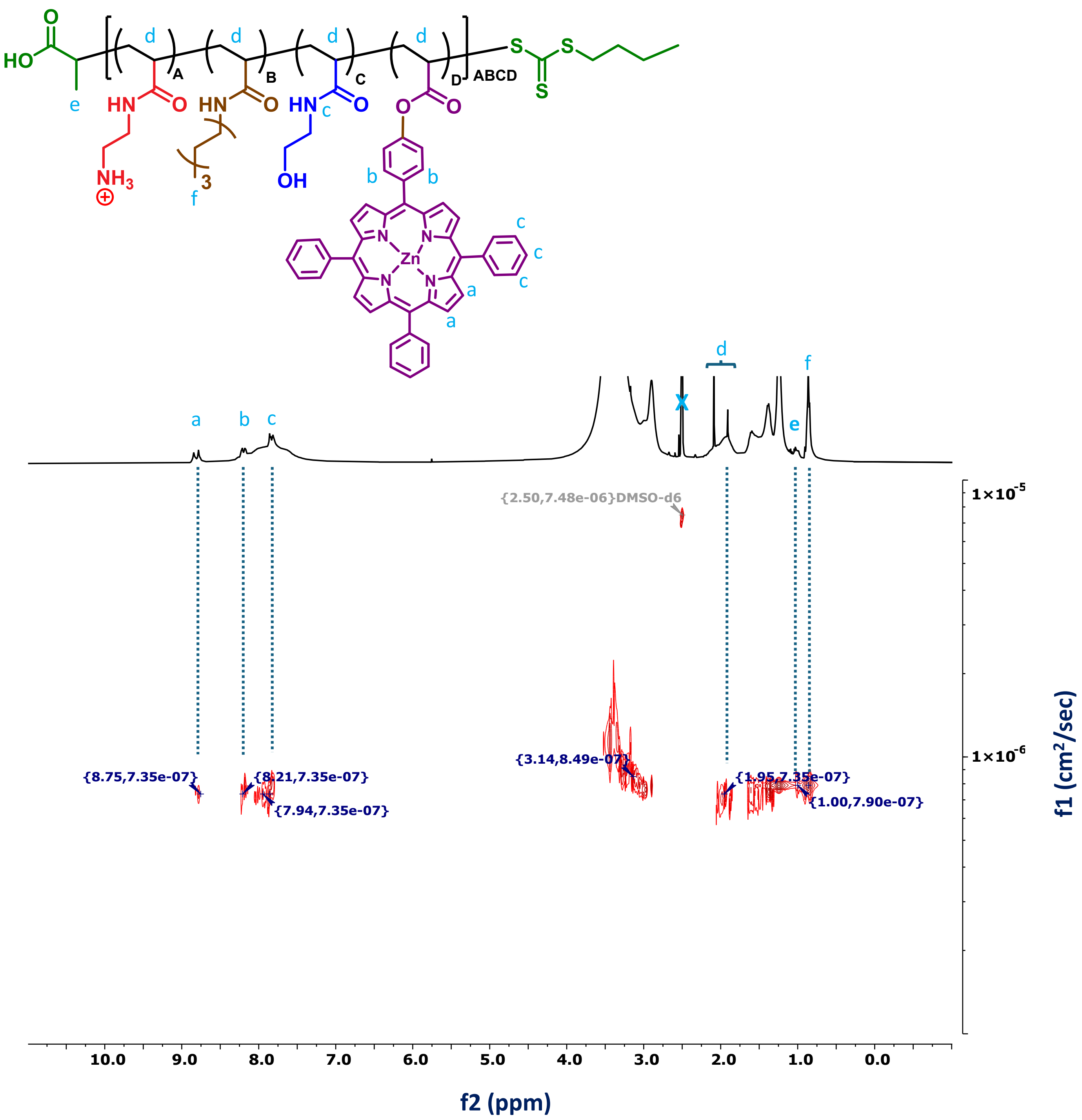

Figure S12. ^{1}H NMR spectrum of the Boc-deprotected polymer P(ABCD) in DMSO-d₆ (top panel), with peak labels a–f corresponding to the assigned signals in the polymer structure. The pseudo-2D DOSY spectrum (bottom panel) confirms that all resonance signals exhibit a common diffusion coefficient (D ~ 10^{-7} cm^2/s), confirming successful incorporation of monomer D and indicating the absence of residual low-molecular-weight species or unreacted components. ***Note***: The blue cross in the ^{1}H NMR spectra indicate signals attributed to DMSO-d_6 and water solvent, which are overlapped with polymer proton signals at 3.3 ppm.

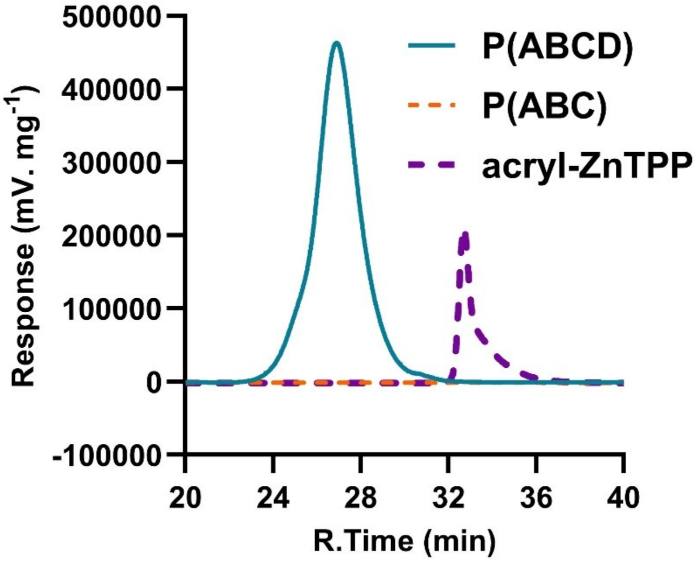

Figure S13. UV-SEC traces (using λ = 420 nm) of P(ABCD), P(ABC), and Acryl-ZnTPP monomer.

Note: P(ABCD) shows a clear absorption signal at 420 nm, confirming the incorporation of acryl-ZnTPP into the polymer chain. In contrast, P(ABC) exhibits no detectable signal at 420 nm, consistent with the absence of acryl-ZnTPP units. The acryl-ZnTPP monomer displays a UV–SEC trace at a higher elution time, showing its lower molecular weight relative to the polymer.

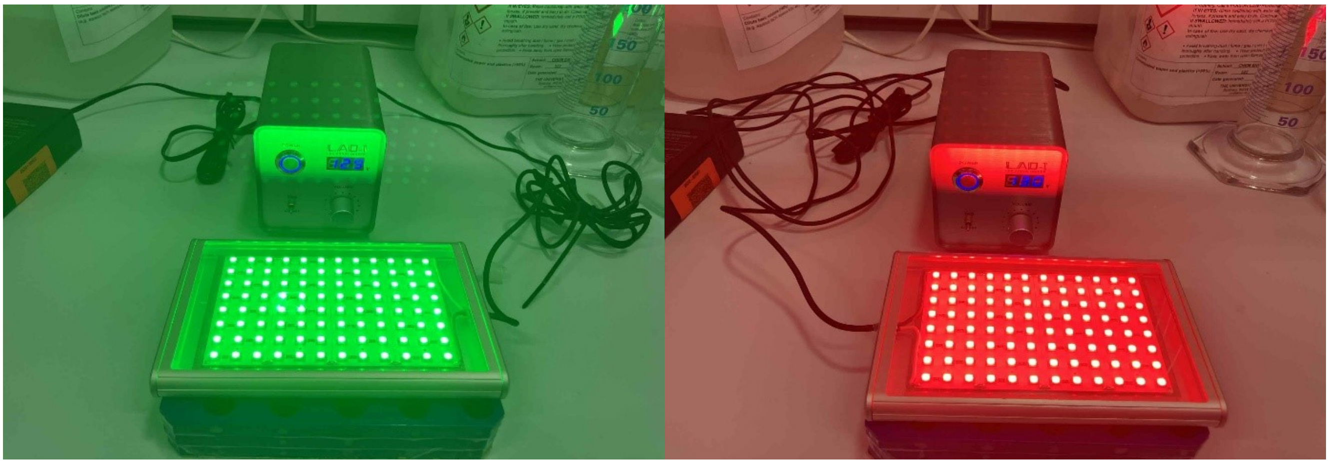

Figure S14. Light photoreactor setup: green light (left) and red light (right).

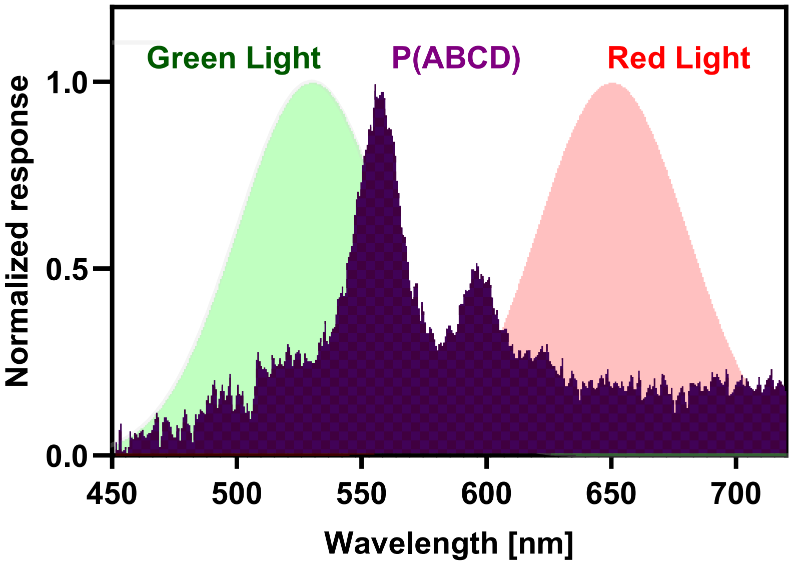

Figure S15. UV-visible absorbance spectra of P(ABCD) and simulated primary spectra. UV-visible spectrum of P(ABCD) compared with simulated primary colors (green, red) plotted as functions of wavelength. The simulation spectra display Gaussian absorption peaks centered at ~530 nm (green) and ~650 nm (red).

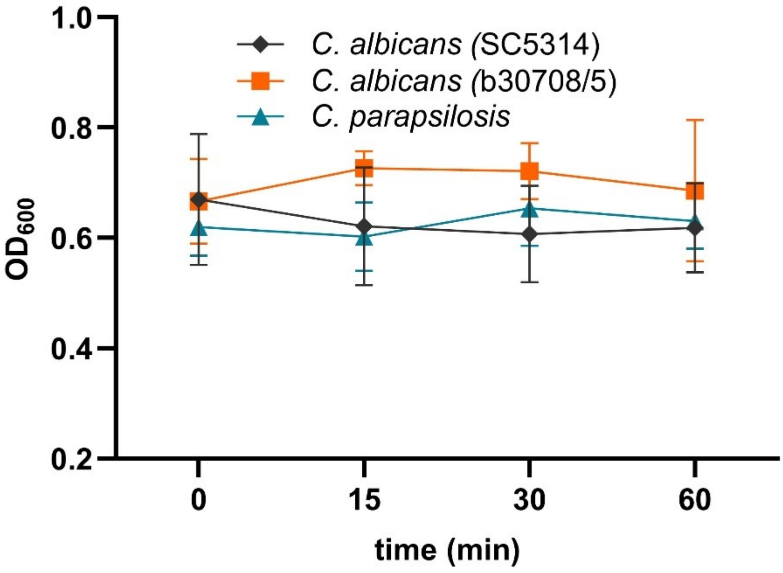

Figure S16. Phototoxicity test under green light irradiation.

Note: Cell growth in a 96-well plate was monitored by measuring optical density at 600 nm (OD_{600}) after exposure to a green LED (~530 nm, 3.45mW/cm², 2 cm distance) for 0, 15, 30, and 60 min. After light treatment, the plates were incubated under standard growth conditions (35 °C) for 24 h. The experiment was performed in triplicate, and the mean values are presented with error bars representing the standard deviation.

Table S1. Comparison of P(ABCD) antifungal activity with and without ROS scavenger treatment.

Fungal strains			***Scavengers***			
	**none**	**none**	**DMSO**	**AscA**	**Mannitol**	**NAC**
	**Irradiation 60 min**					
	-	+	+	+	+	+
	**MIC_{90} (µg mL^{-1})**					
C. albicans SC5314	32	4-8	32	16	32	32
C. albicans b30708/5	256	32	>64	>64	>64	>64
C parapsilosis ATCC 22019	128	16	>64	>64	>64	>64

Note: The MIC_{90} (µg mL^{-1}) values represent the minimum inhibitory concentration required to inhibit at least 90% of *Candida* species growth. The marks (+) were performed under green light (3.45 mW/cm²) for 60 min. Reactive oxygen species (ROS) scavengers used in this assay included Dimethyl sulfoxide (DMSO), Ascorbic acid (AscA), D-Mannitol, and N-Acetyl-L-cysteine (NAC), each at a concentration of 25 µ*M*. Control test confirmed that all scavengers exhibited no measurable antifungal activity at concentrations up to 100 µ*M* (MIC > 1024 µ*M*).

References

1. Gmedhin, H.; Schaefer, S.; Corrigan, N.; Wu, P.; Gu, Z.; Lenardon, M. D.; Boyer, C., Effect of defined block sequence terpolymers on antifungal activity and biocompatibility. *Macromol. Biosci.* **2025,** *25* (4), 2400429.

2. Judzewitsch, P. R.; Corrigan, N.; Wong, E. H. H.; Boyer, C., Photo-enhanced antimicrobial activity of polymers containing an embedded photosensitiser. *Angew. Chem. Int. Ed.* **2021,** *60* (45), 24248-24256.

3. Gillum, A. M.; Tsay, E. Y. H.; Kirsch, D. R., Isolation of the *Candida albicans* gene for orotidine-5′-phosphate decarboxylase by complementation of *S. cerevisiae* ura3 and *E. coli* pyrF mutations. *MGG* **1984,** *198* (1), 179-182.

4. MacCallum, D. M.; Castillo, L.; Nather, K.; Munro, C. A.; Brown, A. J.; Gow, N. A.; Odds, F. C., Property differences among the four major Candida albicans strain clades. *Eukaryotic cell* **2009,** *8* (3), 373-387.

5. Briand, D.; Dubreucq, E.; Galzy, P., Functioning and regioselectivity of the lipase of *Candida parapsilosis* (Ashford) langeron and talice in aqueous medium: New interpretation of regioselectivity taking acyl migration into account. *Eur. J. Biochem.* **1995,** *228* (1), 169-175.

6. CLSI, *Reference method for broth dilution antifungal susceptibility testing of yeasts,Clinical and* Laboratory Standards Institute, 4th ed., Wayne, PA. 2017.

7. Cantón, E.; Pemán, J.; Viudes, A.; Quindós, G.; Gobernado, M.; Espinel-Ingroff, A., Minimum fungicidal concentrations of amphotericin B for bloodstream Candida species. *Diagn. Microbiol. Infect. Dis.* **2003,** *45* (3), 203-206.

8. Judzewitsch, P. R. Z., Lily. Wong, Edgar H. H.; Boyer, C., High-throughput synthesis of antimicrobial copolymers and rapid evaluation of their bioactivity. *Macromolecules* **2019,** *52* (11), 3975-3986.

9. Amorim, C. F.; Iglesias, B. A.; Pinheiro, T. R.; Lacerda, L. E.; Sokolonski, A. R.; Pedreira, B. O.; Moreira, K. S.; Burgo, T. A. L.; Meyer, R.; Azevedo, V.; Portela, R. W., Photodynamic inactivation of different *Candida* species and inhibition of biofilm formation induced by water-soluble porphyrins. *Photodiagn. Photodyn. Ther.* **2023,** *42*, 103343-103343.
